# Comprehending Meningioma Signaling Cascades Using Multipronged Proteomics Approaches & Targeted Validation of Potential Markers

**DOI:** 10.3389/fonc.2020.01600

**Published:** 2020-08-26

**Authors:** Shuvolina Mukherjee, Deeptarup Biswas, Rucha Gadre, Pooja Jain, Nelofer Syed, Julianna Stylianou, Qingyu Zeng, Anita Mahadevan, Sridhar Epari, Prakash Shetty, Aliasgar Moiyadi, Graham Roy Ball, Sanjeeva Srivastava

**Affiliations:** ^1^Proteomics Lab, Department of Biosciences and Bioengineering, IIT Bombay, Mumbai, India; ^2^Centre for Integrative Systems Biology and Bioinformatics, Department of Life Sciences, Imperial College London, London, United Kingdom; ^3^Division of Brain Sciences, Department of Medicine, Imperial College London, London, United Kingdom; ^4^Department of Neuropathology, Human Brain Tissue Repository (Brain Bank), NIMHANS, Bengaluru, India; ^5^Department of Pathology, Tata Memorial Centre, Mumbai, India; ^6^Department of Neurosurgery, Tata Memorial Centre, Mumbai, India; ^7^School of Science and Technology, Nottingham Trent University, Nottingham, United Kingdom

**Keywords:** label-free proteomics, meningioma tissue, patient-derived primary cell line, RT^2^ PCR arrays, Integrin Linked Kinase inhibition, Integrin pathway, PI3K-Akt pathway

## Abstract

Meningiomas are one of the most prevalent primary brain tumors. Our study aims to obtain mechanistic insights of meningioma pathobiology using mass spectrometry-based label-free quantitative proteome analysis to identifying druggable targets and perturbed pathways for therapeutic intervention. Label-free based proteomics study was done from peptide samples of 21 patients and 8 non-tumor controls which were followed up with Phosphoproteomics to identify the kinases and phosphorylated components of the perturbed pathways. *In silico* approaches revealed perturbations in extracellular matrix remodeling and associated cascades. To assess the extent of influence of Integrin and PI3K-Akt pathways, we used an Integrin Linked Kinase inhibitor on patient-derived meningioma cell line and performed a transcriptomic analysis of the components. Furthermore, we designed a Targeted proteomics assay which to the best of our knowledge for very first-time enables identification of peptides from 54 meningioma patients via SRM assay to validate the key proteins emerging from our study. This resulted in the identification of peptides from CLIC1, ES8L2, and AHNK many of which are receptors and kinases and are difficult to be characterized using conventional approaches. Furthermore, we were also able to monitor transitions for proteins like NEK9 and CKAP4 which have been reported to be associated with meningioma pathobiology. We believe, this study can aid in designing peptide-based validation assays for meningioma patients as well as IHC studies for clinical applications.

## Introduction

Meningiomas are tumors that arise from the outer layering of the brain; the exact site of origin of these tumors is believed to be the arachnoid villi cells. Studies have revealed that meningiomas make up for nearly 35% of all primary brain tumors (1). Although, relatively indolent meningiomas can cause comorbidities including neurological and cognitive disorders. In a study by Stafford et al. via a retrospective study they pointed out that even people with WHO Grade I meningiomas had issues for long term survival owing to impaired cognition (2). With the advent of genomics platforms, the implications of mutations in NF2 as well as Non-NF2 tumors harboring mutations at TRAF7, KLF4, and AKT1 have been recognized (3). Recent research has also highlighted novel insights into the methylation patterns and have proposed the existence of multiple subtypes of meningiomas based on their epigenetic signatures (4). However, not many proteomics studies have been employed for deciphering the perturbations at the protein level.

Though limited in number, there have been recent studies that have uncovered newer insights into the way the tumors behave using several proteomics approaches (5–8). We earlier reported an elevation in the levels of AHNAK, Gelsolin, S100 family of proteins, and several other proteins like CKAP4 that were specific to particular grades of meningiomas (9). Several of these candidates which earlier identified via our quantitative proteomics study were also found to be involved in meningioma pathobiology via the current study and independent studies on larger patient cohorts as well (7, 10).

Therefore, one of the major aims of the current study was to further identify protein markers that can in addition to existing histopathology studies point out cases wherein the patients might need closer follow-up. While contemporary studies have investigated peripheral proteomic alterations of meningiomas; we have employed extensive proteomic and meta-analysis to provide a comprehensive landscape of the protein that has emerged from various studies to provide a panel of proteins that can be screened across a larger cohort of meningiomas via targeted proteomics approach (7, 9). As a first-time attempt, we have developed targeted proteomics-based Selected Reaction Monitoring (SRM) Assays toward clinical translation employing a patient-derived spectral library. This enabled validation of peptides of several candidate biomarkers like VIM, ANXA2, AHNAK, TS101, and CLIC1 from the surgically resected meningioma tissues and control arachnoid regions.

Furthermore, several studies have shown prominent involvement of the Integrin and Focal adhesion pathway in meningiomas including Focal Adhesion Kinase (FAK), ERK. Furthermore, approaches that have inhibited FAK *in vivo* models have shown promising results in terms of inhibition of tumor growth (11). From our current and previous study (9), we have identified major perturbation in components of the Integrin pathway namely ITGAM, ECM2, FBLN1, indicating that there is a strong possibility of involvement of ECM members in meningioma pathobiology. Despite playing a crucial role in recruiting several growth factors as well as engaging in crosstalk with several signaling cascades; Integrins, however, are not known to have any intrinsic enzymatic activity. Many of the signaling aspects of the Focal adhesion junction is modulated via the kinases namely the FAK and the Integrin Linked Kinase (ILK) (12, 13). Thus, inhibition of FAK and ILK has emerged as a promising juncture for therapeutic intervention (14–16). An in-depth mapping of the altered signaling cascades and networks of the identified protein candidates using a plethora of approaches including PTM analysis, machine learning as well as *in silico.* the analysis revealed alterations in Focal adhesion, PI3K-Akt and several components that are downstream effectors of ILK. A previous study using meningioma cell lines reported the efficacy of ILK inhibition in meningiomas (16). To explore whether inhibition of ILK can influence the protein candidates and dysregulated pathways emerging from our study, in this current study we have treated patient-derived primary cell lines with an ILK inhibitor (Cpd22) to assess transcriptional level alterations.

Herein, we present a comprehensive proteomic profile of meningioma patients aimed to look at protein markers for the identification of a panel of protein that can be screened in a large patient cohort using targeted proteomics assay as well as immunohistochemistry. We further provide the first report of SRM assays in meningiomas in nearly 50 patients. Our study also investigates network-level perturbations via machine learning approaches, phosphoproteomics to map perturbed pathways. For the identification of a potential adjuvant therapeutic adjunct in meningiomas where the tumor is either aggressive, recurrent or is unamenable to conventional surgery owing to tumor location; we have assessed the implication of ILK inhibition in meningioma cell lines.

## Materials and Methods

### Sample Collection

All samples were collected following the IEC (Institute Ethics Committee, IIT Bombay) and IRB (Institute Review Board, Tata Memorial Hospital, Mumbai). This study was approved as part of an institutional review board (ACTREC-TMC IEC No.149, Advanced Centre for Treatment, Research and Education in Cancer and Tata Memorial Centre) following approved guidelines. Patients with radiologically suspected meningiomas were enrolled after giving written informed consent. Surgically resected tissues were procured from patients diagnosed to be meningiomas by radiology. Tumor tissues were flash-frozen in liquid nitrogen (Supplementary Table 1).

### Protein Extraction and Digestion

Global proteomic profiling/shotgun proteomics was performed across MG1 (*n* = 10), MG II (*n* = 11), MGIII (*n* = 2), Normal dura mater (*n* = 4), and Arachnoid mater (*n* = 4). Protein extraction was done using Urea buffer (8 M Urea, Tris–HCl buffer) with the addition of Phosphatase inhibitor cocktail (SigmaAldrich^®^, United States) as mentioned in the protocol by CPTAC Investigators (17). In brief, tumor tissue was washed with 1X PBS and cut (around 75 mg) tissue lysis was performed with sonication followed by bead milling at 90 s for 3 cycles. The lysates were centrifuged at 12000 r.p.m at 4 degrees for 15 min to clear the debris, the supernatant was quantified using 2D-Quantification kit (Bio-Rad, United States) and 100 μg of protein was digested using Trypsin (Pierce, Thermo Fisher Scientific, United States) for 16 h at 37°C Followed by vacuum drying the peptides and reconstitution with 0.1% Formic Acid. The peptides were quantified using Thermo plate reader using Scope’s method 1 μg of the peptide was used for the LC-MS/MS run.

### Mass Spectrometry Based Global Proteomic Analysis of Meningioma Patient Cohort and Examination of the Phosphopeptides From the Meningioma Patient Cohort

All patient samples and non-tumor samples were run in the Q-Exactive Orbitrap Mass Spectrometer (Thermo Fisher Scientific, United States) using a gradient of 0.1% FA and Acetonitrile for 240 with blanks after every sample. The scan range was set from 350–1700 m/z and the resolution was set to 17,500. Phosphoproteomic enrichment was done using the TiO_2_ enrichment column (Pierce, Thermo Fisher Scientific, United States). In brief protein extraction of surgically resected tissues (MGI, *n* = 6, MGII, *n* = 10, and MGIII, *n* = 2) were done from 18 patient samples, around 300 μg of protein was digested using Trypsin (Pierce, Thermo Fisher Scientific, United States), followed by peptide level enrichment using TiO_2_ enrichment column, enriched peptides were further cleaned up using graphite clean-up kit.

### Data Acquisition and Analysis by Proteome Discoverer 2.2

All.raw files acquired were analyzed using Proteome Discoverer Version 2.2 using 1% FDR for both the protein as well as peptides. Static modification for the label-free run was set to Carbamidomethyl and for the phosphoproteomics run it was the same with S, T, and Y selected for the variable modification. The database used for protein annotation was Uniprot using Homo sapiens data as background. Additionally, the search engines used were MASCOT and SEQUEST. Total peptide intensity was used for data normalization post missing value imputation on the procured abundances. Metaboanalyst was used for visualization of the heatmaps (18). (Supplementary Figure 1: Parameters of analysis in Proteome Discoverer 2.2).

### Generation of Transition List for SRM Assay Using Skyline

The transition list of proteins was prepared using the Skyline (Version 4.2) (19). The spectral library generation was done using the raw data as generated from the meningioma patients via the label-free proteomic analysis. The “precursor charges” used were 2, Ion charges used were 1, 2; y ions were selected. Precursor and product masses were set to be “Monoisotopic.”

### SRM Assay for Differentially Expressed Proteins

The transition list of differentially expressed proteins was prepared using Skyline^®^. Transitions of specific proteins were optimized along with the LC parameters. All runs were performed using nano-LC mode. The assay was performed in Triple Quadrupole Instrument (TSQ, ThermoFisher Scientific, United States) Peptides (1 μg) was reconstituted in 10 μl 0.1% FA. The peptides were then run in a gradient comprising of solvent A (0.1% FA) Solvent B (80% ACN with 0.1% FA) for 45 min per run. The flow rate was maintained at 300 nl/min and the column used was ES 803 Easy spray pepmap C18 column. The acquisition was done using TSQ Altis™ Triple Quadrupole Mass Spectrometer (Thermo Fisher Scientific, United States) using a method duration of 45 min.

### Cell Culture and Inhibition Assay

The primary cells were procured from the Tissue Bank, Imperial College London. The primary cells were seeded from surgically resected tissues of meningioma patients. The cells were passaged prior to freezing in liquid Nitrogen for long term storage. The cells were taken out and revived in DMEM-F12 media. However, owing to the slow growth (doubling time was nearly 50 h) the cells were moved to Waymouth media supplemented with 20% FBS. For the inhibition assay cells were passaged in 6 well plates in triplicates and treated with 2.5 μM of ILK inhibitor namely Cpd22, Merck Millipore. The cells were seeded in two batches “Untreated” (No drug) and “Treated” (With Drug). The treatment was carried out for 24 h. Post-treatment the cells were pelleted down and further taken forward for RNA extraction.

### RNA Extraction and cDNA Synthesis

Cells were lysed using RLT buffer and the RNA extraction was done using the Qiagen^®^ RNA Easy extraction Kit (Catalog No: 74104) as per the kit protocol. Briefly, cells were lysed and then mixed with 70% ethanol followed by loading on mini-spin columns. The RNA yield was determined using a spectrophotometer Two-step cDNA synthesis was carried out using the MMLV RT cDNA synthesis kit.

### RT^2^PCR Using Pathway Specific Arrays

The RT^2^ PCR was done using the Qiagen RT^2^ PCR array of two pathways namely the Cytoskeletal Regulators and Akt Pathway. The experimental set up included two conditions treated vs untreated cells (Meningioma Primary Cell lines of MGI tumor origin) treated with Cpd22 (Integrin Linked Kinase Inhibitor, Merck Millipore). Mature RNA was isolated using an RNA extraction kit according to the manufacturer’s instructions. RNA quality was determined using a spectrophotometer and was reverse transcribed using a cDNA conversion kit. The cDNA was used in the real-time RT^2^ Profiler PCR Array (QIAGEN, Cat. no. PAHS-088Z) in combination with RT2 SYBR^®^Green qPCR Mastermix (Cat. no. 330529). CT values were exported to an Excel file to create a table of CT values. This table was then uploaded on to the data analysis web portal at http://www.qiagen.com/proteinglobe. Samples were assigned to controls and test groups. CT values were normalized based on the automatic selection from the HKG panel of reference protein (Supplementary Data 8).

### Pathway Analysis and PPI: Protein-Protein Interaction Analysis

We performed functional annotation clustering analysis for ANOVA passed protein using DAVID (20, 21). Parameters used were custom classification stringency setting; similarity term overlap = 5, similarity threshold = 0.95, initial group membership = 3, final group membership = 3, multiple linkage threshold = 0.5, EASE score = 1.0, and Benjamini-Hochberg correction was applied. Enrichment score was taken into account to narrow down the cluster count. Further, investigation of complex interaction and prediction of pathways among the significant proteins in MG1 vs MG2 were done using Reactome.org (22) and KEGG Database (23). A multi-functional online software NetworkAnalyst 3.0 (24) was used to analyze the protein list for constructing the visualized PPI network. Kinome analysis was performed in kinhub.org with the kinases identified from the dataset. Additionally, REVIGO was used for visualizing the GO terms associated with the phosphopeptides (25). For investigating the effect of Cpd22 on biological pathways of the meningioma cell lines Reactome.org (22) and KEGG (23) were used. To understand the effect of the inhibitor on pathways like PI3K-Akt and Focal Adhesion, the selected pathway entities were extracted from NCI nature (26), KEGG 2019 and Reactome 2019 databases and the list of significant proteins were mapped. Furthermore, the information from the analysis was taken to build a biological pathway model (Supplementary Data 5, 6, 8, 9).

### MLP and Neural Network-Based Mining for Identification of Key Drivers

Multilayer perceptron (MLP), a 3-layered parsimonious MLP architecture using sigmoid activation functions with a feed-forward – backpropagation learning algorithm was applied to model protein-protein interactions between proteins perturbed in meningiomas as identified via proteomic analysis. ANOVA passed proteins were taken for the Low-Grade vs High-Grade comparison. To define an interaction map for the proteins, the summed weights of the trained ANN model, leading from a given input to a given output, were used to illustrate and score the interaction between proteins (27). The Pearson correlation coefficient r with a cut-off value of 0.7 was implemented in the algorithm to remove the least significant interaction scores. Monte-Carlo cross-validation (MCCV). To prevent the ANN model from being over-trained, an MCCV strategy was applied as follows. The algorithm was coded in C and empirical work on the dataset was presented in the subsequent section. Visualization of interactome network maps The Cytoscape software platform (version 2.8) for molecular interaction display was used in this study. The above approaches were adapted and modified for the current data set from (27–29) (Supplementary Data 4B).

## Results

### Clinical Parameters

The patients were examined via MRI for determining the tumor location. Additionally, surgically resected tissues were examined by standard histopathology as per the WHO guidelines for assigning the grades. For the Global proteomics study, 10 MGI (Benign) and 11 MGII (Atypical) cases were taken forward (Supplementary Data 1).

### Label-Free Quantification of Surgically Resected Meningiomas to Identify Altered Proteomic Signatures

**FIGURE 1:**
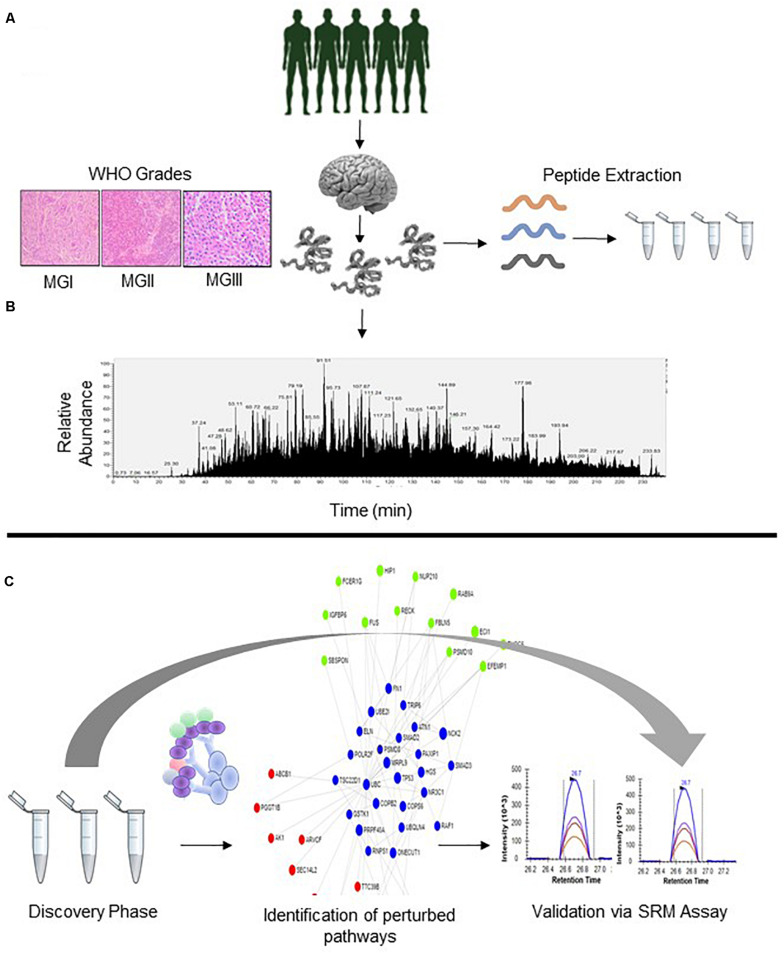
Overall Schematic of Proteomic Analysis of Meningioma Patients. **(A)** Patients suspected to have meningioma **(B)** Analysis of WHO Grade-specific proteomic alterations; patient segregation based on WHO classification **(C)** Gene Ontology, PPI, and Pathway mapping and development of SRM Assays for the analyzed proteins.

Patients with radiologically suspected meningiomas were accrued for the study. Patients were assigned WHO Grades namely MGI, MGII, and MGIII post histopathological examination. The information of the accrued patients is mentioned in Supplementary Table 1. Individual proteomic analysis of the patients was performed in LC-MS in Orbitrap Q-Exactive which enabled the identification of nearly 5659 Master Proteins with 1% False Discovery Rate. Further filtering based on ≥2 unique peptides enabled the identification of nearly 4600 proteins with ≥2 unique peptides across the cohort with high confidence. Further, Log_2_ transformation of the obtained abundances was performed which were taken forward for further statistical analysis (Figure 1, Table 1, and Supplementary Data 2, and Supplementary Figure 1).

**TABLE 1:** Key candidate proteins with role in meningioma pathobiology, monitored peptides via SRM Assay.

Protein	Avg. Abundance (Log Scale)			Peptides Monitored via SRM Assay	Role in meningiomas/Reported via other studies	Phosphosite identified	Influence on signaling pathway(s)	Inhibitors/Therapeutic intervention points
	NT (*n* = 8)	MGI (*n* = 10)	MGII (*n* = 11)					
VIM (P08670)	34.7	36.0	35.2	R.DNLAEDIMR.L K.LQEEMLQR.E R.QDVDNASLAR.L	Phosphorylated form of Vimentin ascribed in non-infiltrative meningiomas (39, 40)	Phospho [S7; T20; S22; S25; S26; S34; S39; S51; Y53; S55; S56; S72; S73; S83; S87; S214; S226; S325; S339; S409; S412; S419; S420; T426; S430; S438; T458; S459]	Vimentin a component of extracellular matrix also is a substrate for multiple kinases (41)	Withaferin-A (WFA) (41)
ANXA2 (P07355)	31.3	33.1	32.8	K.AYTNFDAER.D R.QIAFAYOR.R K.TPAQYDASELK.A	Annexin A2 as well as key interactor S100A10 has been reported to be elevated in meningiomas (42–44)	Phospho [S12; Y24; S26; S89; S92; Y188; Y199]	Annexin A2 is a multifunctional protein mediating actin remodeling, membrane assembly, signal transduction, etc. (45, 46)	Tri-Substituted 1,2,4-triazoles inhibitANXA2-S100A10 interaction (42)
CKAP4 (Q07065)	26.6	28.5	28.7	R.LPPQDFLDR.L K.ASVSQVEADLK.M R.TAVDSLVAYSVK.I	CKAP4 has been seen to be aberrantly expressed in meningiomas via multipronged approaches including IHC (7)	Phospho [S460; S461]	CKAP4 mediates activation of the PI3K-Akt pathway.	
CLIC1 (O00299)	25.7	27.5	27.3	K.IGNCPFSQR.L K.LAALNPESNTAGLDIFAK.F R.GFTIPEAFR.G	Reported to be upregulated in meningiomas especially in atypical and anaplastic types (7)	NA	Regulates MAPK/Akt pathways (47)	Biguanide-related drugs (47)
EIF4G1 (Q04637)	24.2	25.7	26.7	K.QVTVLAIDTEER.L K.GVIDLIFEK.A K.VEYTLGEESEAPGQR.A	Critical component of translational initiation. Known to function as an oncoprotein in several cancers (44)	Phospho [S1185; S1187]	Translational machinima as well as influence PI3K/AKT/mTOR pathway	Silvestrol has ha inhibiory effects on EIF family in meningioma cell lines (48)

*Details mentioned in Supplementary Data 2, 6, 9A.*

### Evaluating Grade-Wise Markers in Meningioma Patients, Correlation With Histopathological Features

**FIGURE 2:**
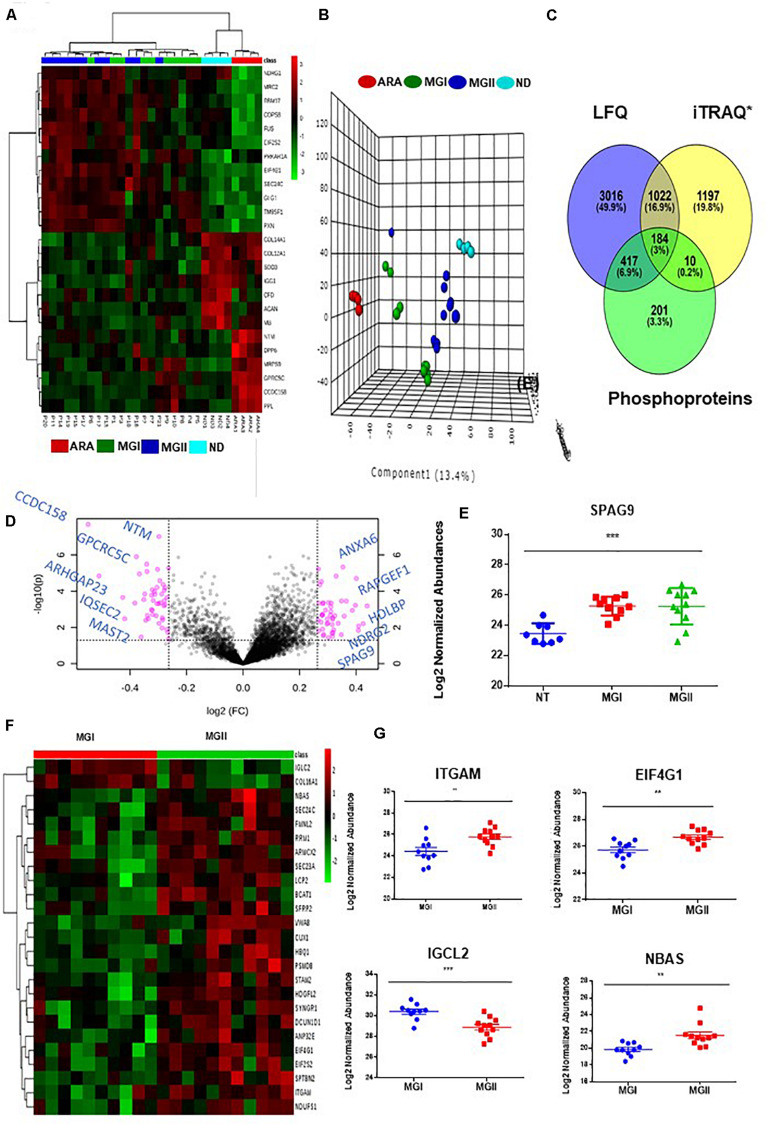
Identification of meningioma subtypes using proteomics and examining the perturbed networks. **(A)** Unsupervised clustering (Distance measure: Euclidian; Clustering algorithm: Ward) reveals distinct subgroups meningiomas **(B)**. Principle Component Analysis reveals the segregation of MGI, MGII, Dura, and Arachnoid; few MGI cases seemed to exhibit proteomic profiles as that of the atypical tumors **(C)** Cross comparison of Meningioma Label-free proteomic data set from individual patients with quantitative grade-wise proteomics study and PTM analysis. **(D)** Volcano plot (MG vs Non-tumor controls) Log FC ≥ 1.2. **(E)** Log 2 Normalized abundance across grades and non-tumor control of SPAG9 **(F)** Supervised Hierchial Clustering reveals grade-specific protein profile in MGI vs MGII cases **(G)** Key altered proteins between MGI vs MGII (*t*-test passed; *p* < 0.05).

**FIGURE 3:**
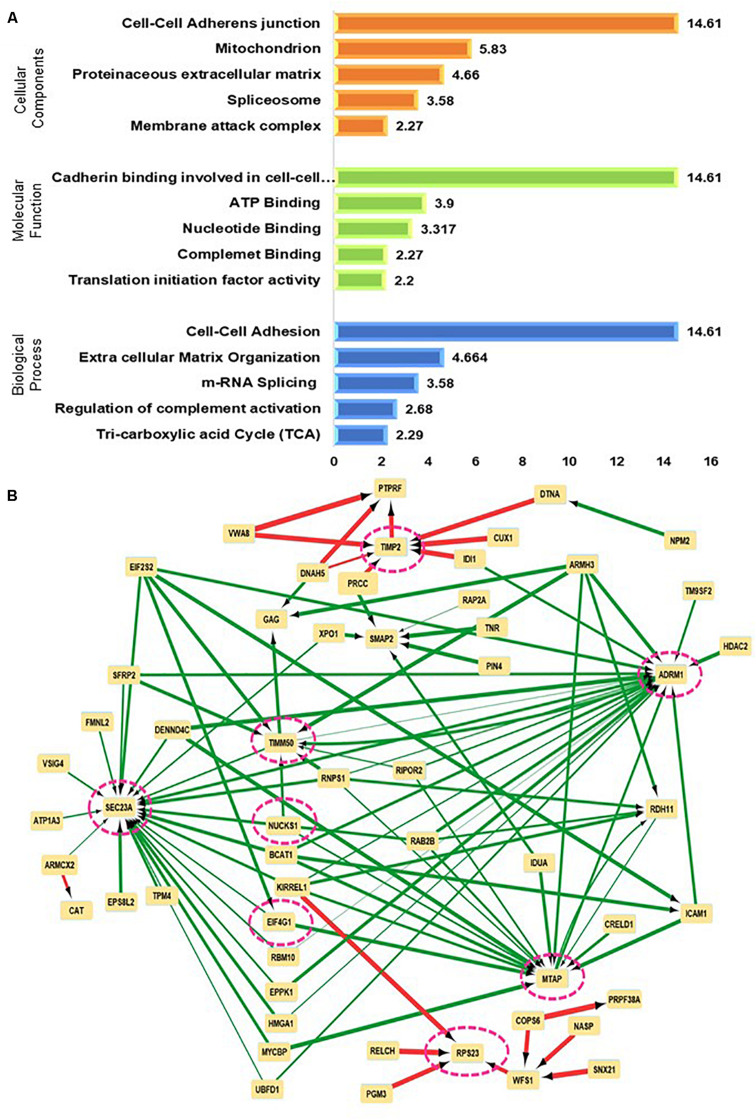
Mapping perturbed pathways and networks from grade-wise meningioma tissue comparisons **(A)** Functional enrichment test on significant proteins emerging from comparisons of different grades of meningioma **(B)** Neural networking of significantly altered proteins using lower grade vs higher-grade meningiomas as predicted via a Multilayer perceptron (MLP) neural network architecture.

The comparison of the significant proteins as obtained post ANOVA test was further subjected to Unsupervised clustering which revealed that there were three clusters (Figure 2A). As per the heatmap it is visible that NDRG1, MRC2, and COPS8 is highly abundant in the higher grades of meningiomas whereas more abundances of COL14A, COL12A1 were found in the non-tumor controls (Figures 2A,B). Furthermore, a volcano plot analysis of the entire dataset with MG vs Non-tumor controls revealed significant upheaval in proteins like SPAG9, NDRG2 (Figure 2D). Comparison of MGI (*n* = 10) and MGII (*n* = 11) enabled the identification of nearly 230 proteins with high confidence that were significantly (*p* ≤ 0.05) altered in MGI vs MGII. NBAS, TIMP2, and NCK1 were few of the key proteins found to be altered in Atypical meningiomas (Figures 2F,G and Supplementary Data 3). Furthermore, correlation of the proteomic and histopathological subgroups was found out in the patient cohort with WHO classified patients that were annotated MGI; however, had bone invasion clustered with the Atypical patients indicating the possibility of intratumor heterogeneity and a need for closer follow up of these patients (Figure 2A). We probed the 672 proteins that have significantly passed ANOVA for the Gene Ontology. Grade-wise comparisons on MGI (benign) vs MGII (atypical) meningiomas revealed prominent involvement of TIMM50, SEC23A, MTAP, RPS23, and ADRM1 based on the concordance analysis of highly ranked features from across 20 Machine Learning randomized iterations obtained via the methods of the Ball group (28) (Figures 3A,B and Supplementary Data 4B, 5).

**FIGURE 4:**
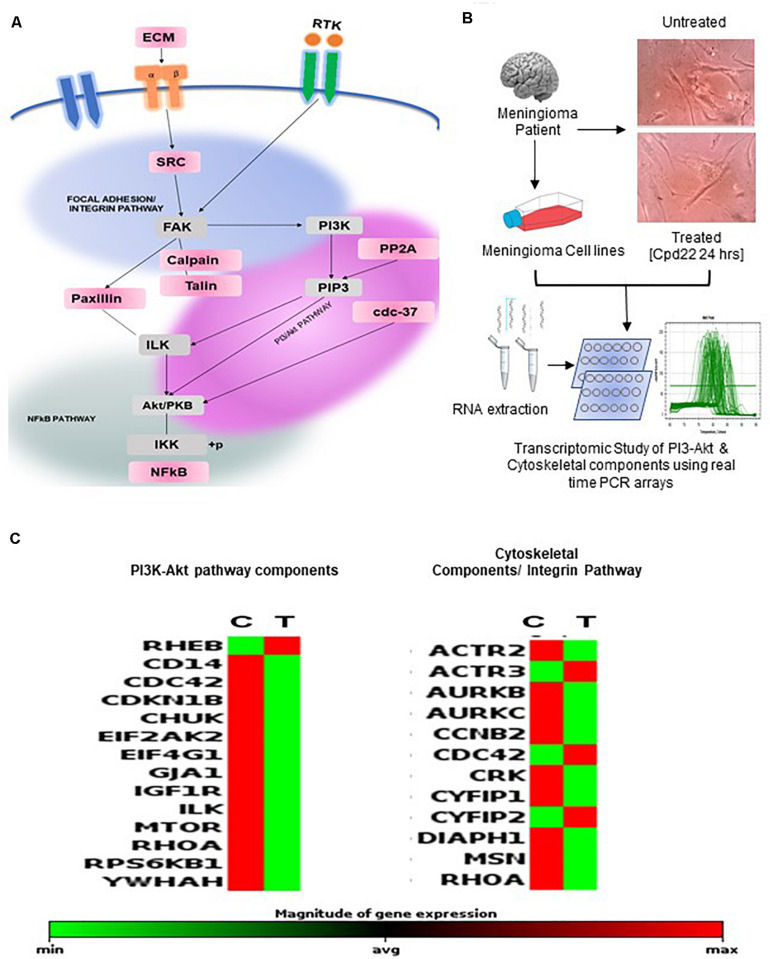
Assessment of perturbations of Integrin and PI3K-Akt pathway in meningiomas: **(A)** Hypothesis of possible crosstalk among PI3-Akt, NFkappaB, and Focal adhesion pathways **(B)** Workflow depicting Patient-derived primary cell line which was treated with 2.5 μM of ILK inhibitor and examined for alterations in the transcriptome with a specific focus on PI3-Akt pathway and Cytoskeletal Regulators **(C)** Alterations of PI3K-Akt and Cytoskeletal components as derived from the via transcriptomic analysis.

The functional annotation clustering based on Molecular Function; Biological Process, and Cellular component provides 135 clusters taking the 672 proteins into account which were significant in meningiomas vs non-tumor controls. The top hits in terms of Biological Processes were, cell-cell adhesion (*p*-value = 1.39E-13, ES = 14.61) and Extracellular Matrix Organization (*p*-value = 1.53E-06, ES = 4.664). In the ECM clusters, a hub of proteins that were exclusively found to be related to collagen biosynthesis was COL4A4, COL6A2, COL14A1, COL16A1, and COL18A. Several members of extracellular matrix remodeling and Integrin signaling like ECM2, LUM, ITGB4, ITGM, and Fibulin family were also mapped. The proteins of these clusters were related to the PI3K-Akt signaling pathway and Focal adhesion. On the other hand, in cell-cell adhesion Annexin A1, Annexin A2, the hub of the actin-binding protein, and proteins related to translational machinery were also found (Supplementary Data 4A). The Gene Set Enrichment analysis of overall identified significant proteins revealed the involvement of Focal Adhesion, PI3K-Akt, Hippo, VEGF, and Ras signaling pathways with possibilities of crosstalk among the dysregulated pathways. Figures 3A,B, 4A (Supplementary Data 4A).

### Identification of Phosphorylated Proteins From Meningioma Patients

**FIGURE 5:**
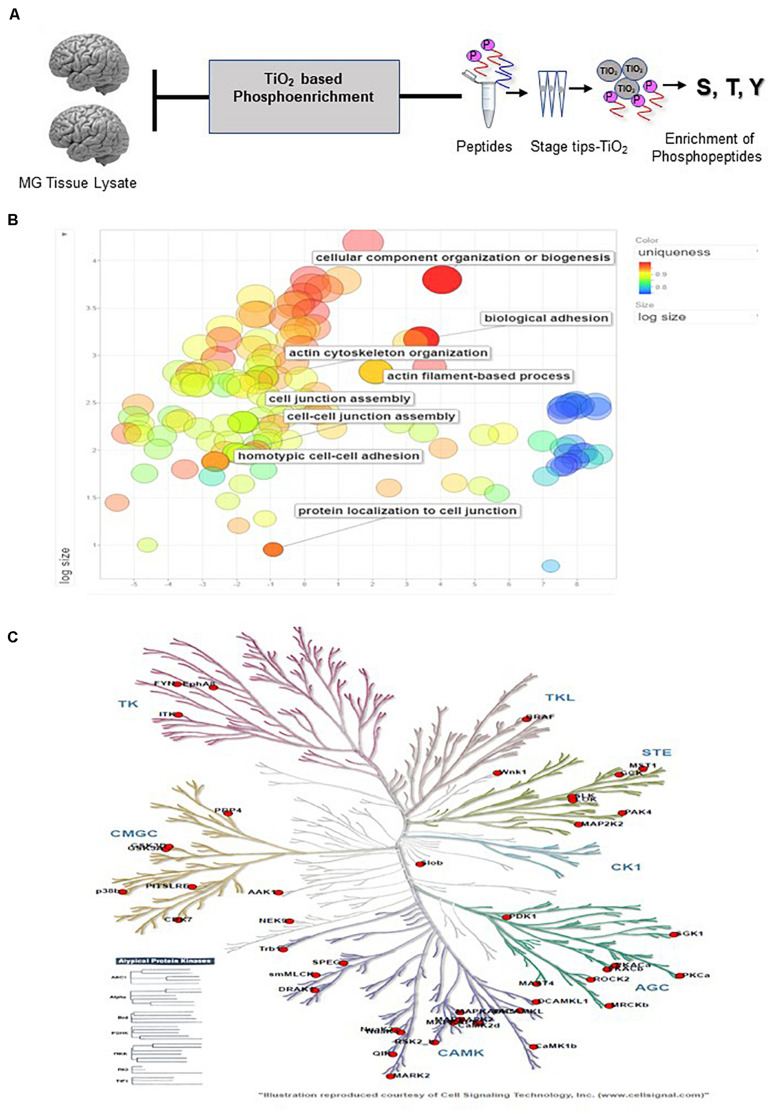
Outcomes of PTM Analysis **(A)** Workflow for enriching the samples for Phosphopeptide enrichment using Titanium dioxide Stage. **(B)** REVIGO Analysis revealed the Gene Ontology-based enrichment of the identified phosphopeptides **(C)** Kinome Analysis performed in http://kinhub.org enabled identification of the Kinome map of the kinases identified via study (Supplementary Data 6).

Investigating the global proteome level alterations also lead to the identification of proteins that were involved in key signaling cascades. To assess the post-translational modification of these proteins we investigated the phosphorylation status in the patient group. Phosphoproteomics was performed using TiO_2_ based enrichment approach on 18 patients to identify the phosphorylation status of the S, T, and Y of the key proteins that were found to be altered in meningioma patients. We were able to identify nearly 812 proteins that had ≥2 unique peptides with high confidence. This study revealed the identification of phosphorylated sites of several proteins that were found to play a role in meningioma pathobiology by several studies including EPB41L2, NDRG2, SPTB, MAGED2, MXRA7, and nearly 51 Kinases which were also mapped further through Kinome Analysis (Table 2, Supplementary Data 6 and Figures 2C, 5A–C).

**TABLE 2:** Key proteins and Kinases Identified via the phosphoproteomic analysis followed by Kinome Mapping and their potential role in meningioma pathobiology (Supplementary Data Table 6).

Protein	Identified Phosphosite	Potential role in meningiomas	Influence on signaling pathway(s)	Points of therapeutic intervention
Q9H1E3 (NUCKS1)	Phospho [S19; S54; S58; S61; S181; T202; S204; S214; T220; S221; T222; S223]	Modulates chromatin structure and key events of replication and transcription. It is known to be phosphorylated by CK2, Cdks, and is increasingly being noted as a cancer biomarker (49). Reported to be upregulated in atypical and anaplastic meningiomas (5)	Influences Insulin mediated signaling, NFkB as well as Myc (49)	miR-142-3p has been reported to inhibit NUCKS1 (50)
Q02952 (AKAP12)	Phospho [S219; S280; S283; T285; S286; S312; S371; T597; S598; S627; S629; S696; S697; S698; S1587]	AKAP12 has been reported as a central regulator of meningioma aggressiveness with a possible role in progression (5).	Known to downplay events linked with metastasis and invasiveness. It acts as an antagonist to VEGF (51)	A few peptide inhibitors and a small molecule inhibitor FMP−API−1 have shown to disrupt the AKAP-PKA (52)
Q9P0K1 (ADAM22)	Phospho [S832; S834; S857]	The ADAM22 gene encodes a protein that interacts with LGI1 and forms a complex. It is known to interact with integrins in the brain as well (53)	ADAM22 is known to interact with NRG1 which in turn phosphorylates AKT (53).	Pidolic acid (53)
P08648 (ITGA5)	Phospho [S127; S128]	Integrin alpha-5/beta-1 (ITGA5:ITGB1) is a receptor for fibronectin and fibrinogen (54).	Associated with cell adhesion, focal adhesion functionalities (55).	Resveratrol (55)
Q96B36 (AKT1S1)	Phospho [S88; S92; S202; S203]	AKT1S1 is a proline-rich substrate of AKT that binds to 14-3-3 protein when phosphorylated	Play a crucial role in mediating mTOR and PI3K-Akt signaling cascades	OSI-027 (56)
Q92597 (NDRG1)	Phospho [S326; T328; S330; S332; S333; T335; S336; S364; T366; S367; T375; T699; T699]	Protein encoded by NDRG1 is involved in p53-mediated caspase activation and apoptosis (54). NDRG2 another family member of the NDRGs is known to play role in meningioma tumor recurrence (54)	Involved in NF-kappa B Signaling PI3K/Akt Signaling (54)	Calcium, Okadaic acid (54)
Q9UN36 (NDRG2)	Phospho [S328; T330; S332; S338]	NDRG2 is thought to act as a tumor suppressor. Loss of NDRG2 and decreased presence at genetic level detected in higher grades of meningiomas (54).	Regulates cyclin D1, exerts anti-tumor effects via activation of p38 MAPK. Modulates cell survival and invasion (54, 57).	Fostamatinib (54)

### Collation of Altered Proteomic Signatures Procured From Individual Patients With Grade-Wise Differential Surrogate Markers

We had previously identified several differentially expressed proteins in various grades of meningiomas using iTRAQ based proteomics study (9); 1206 of the differentially expressed proteins were also found out in the individual label-free proteomic analysis substantiating the prominent roles of these in meningioma pathobiology. Some of the key proteins identified via both the approaches include several regulators and cytoskeletal components like AHNAK, PLEC, FLNB, and DSP; routinely used IHC markers like VIM, S100 and Annexin family proteins were also identified with high confidence (1% FDR, ≥2 unique peptides; Supplementary Data 7).

### Perturbation of Several Integrin Components and Influence of Inhibition of Integrin Linked Kinase in Meningioma Primary Cell Line

The global proteomic analysis of meningioma patients in the current study revealed perturbations in several components of Integrin including ITGAV, ITGB2, ITGA2B, and ILKAP (Supplementary Table 2 and Supplementary Figure 6). Furthermore, comparative analysis of Integrin components with Sharma et al., 2015 (9) revealed perturbations in ILKAP, ITGA6 as well as a kinase named ILK which is a key mediator of the integrin pathway and several downstream cascades. Further *in silico* analysis enabled the identification of major interacting nodes in the Integrin and PI3K-Akt pathways which could be downstream effectors of ILK (Supplementary Data 4A).

The inhibition of primary cell lines of meningioma with ILK inhibitor (Cpd22) was performed in meningioma patient-derived primary cell lines. The transcriptome level alterations in various components of PI3K-Akt and Cytoskeletal modulators were identified via transcriptomics level analysis using pathways specific RT^2^ PCR arrays. Key candidate genes altered in the Control vs Treated were MTOR, RHOA, EIF4G1 from the PI3K-Akt pathway and MSN, CYFIP2, AURKC in the Integrin pathway (Figures 4B,C, Supplementary Table 8, and Supplementary Figures 2–4).

### Corroboration of Altered Protein Signatures to Evaluate Relative Abundance Across Individual Patient Cohort via SRM Assay Employing a Patient-Specific Spectral Library

**FIGURE 6:**
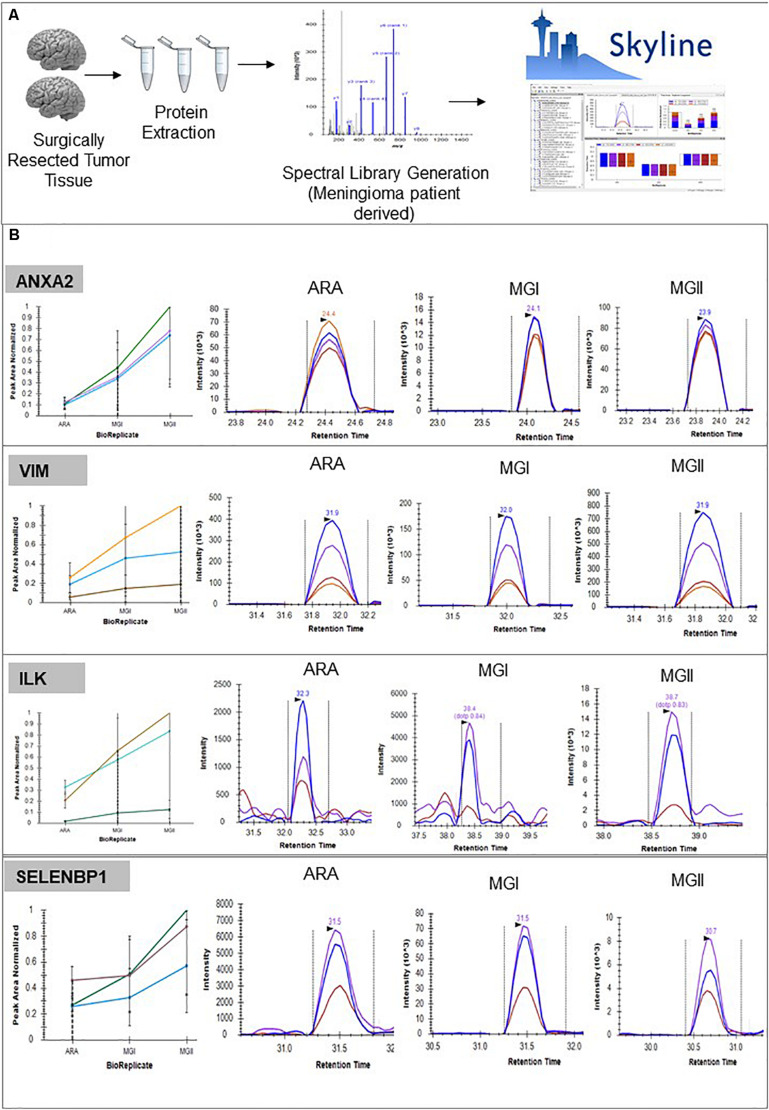
Panel of proteins identified as potential biomarkers. **(A)** Pipeline followed for developing peptide level validation of protein markers from meningioma tissue using targeted proteomics approach including using the global proteome data as a spectral library and using Skyline^®^ for further assay development **(B)** Validation of biomarkers using SRM-based assays. (Details of peptides screened, Peak Areas detailed in Supplementary Data 8, Supplementary Figure 5).

Using the individual patient spectra, we prepared a patient-specific “Spectral Library” for developing a targeted proteomics assay to validate key proteins. We monitored transitions for proteins like VIM, ANXA2, S100 family proteins. Also, we validated targets like AHNAK, tumor-specific proteins like TPD52L2, TS101. Peptides corresponding to NEK9, SELENBP1 which were previously reported as a marker for aggressivity via two independent proteomics study were also optimized and monitored across the meningioma patient cohort (5, 7). We further monitored the levels of PI3K-Akt and Integrin family members like EIF4G1 and CKAP4 (Figure 6, Supplementary Data 7, 9, and Supplementary Figures 5, 6).

## Discussion

Our study employs a plethora of approaches to look into pathway-specific perturbations in meningioma tissue with an emphasis on the Integrin and PI3K-Akt components employing targeted transcriptomics as well as proteomics approaches. To the best of our knowledge, this is the first study that employs targeted proteomics workflow for monitoring peptides from surgically resected tissues in meningioma patients. The protein markers that have emerged from several studies were screened using this approach and thus can be further used for designing peptide-based assays and immunohistochemistry (IHC) based studies for meningioma patients (7, 9, 10).

### Potential of the Label-Free Proteomic Analysis on Identifying Protein Markers and Use of Patient-Derived Spectral Libraries

Meningiomas are one of the most prevalent primary tumors originating from the outer layer of the brain. Several recent studies have highlighted the novel features of these tumors, including alterations at the genetic, epigenetic, and proteomic levels, which has potentiated the possibility of identifying markers that can be used in the clinics for better prognostication of patients (7, 30, 31). There is an imminent need for markers that can be used for prognosis as well as potential targets for therapeutic interventions. The latter is particularly important in cases where the tumors might not be amenable for complete surgical resection or in those cases where a lower grade tumor might have the potential to turn aggressive. In our current study, we have employed label-free proteomics to decipher proteomic alterations in meningioma patients. Using a label-free proteomics approach we identified 7978 Protein groups; 52695 Peptide groups and 1043972 PSMs. The study enabled the identification of nearly 4600 proteins with 1% FDR (≥2 Unique peptides) from surgically resected tumor tissues. Additionally, we have used a “patient-derived spectral library” generated via the global proteomic analysis to develop a targeted proteomic assay workflow for validating several of the altered proteins.

### Protein Markers That Can Aid in the Prediction of Patient Prognosis

We compared the proteomic landscape emerging from the various meningiomas stratified based on the WHO guidelines. The study revealed the identification of 672 proteins that were significantly altered among the meningioma grades and non-tumor controls namely the arachnoid and dura regions and 235 proteins were found to be dysregulated among the MGI and MGII patient cohort indicating the possibility of these proteins to be grades-specific. Additionally, using “unsupervised clustering” we observed that a few cases of even lower grades did segregate with atypical cases. On closer examinations of their histopathological signatures, it randomized that some of the patients had bone invasion. The elevated abundance of proteins like NDRG1, MRC2, and FUS which were found to be higher in the atypical cases in a few of the MGI cases indicates a possibility that these cases might have a chance of recurrence in future and warrants a closer follow up. It is interesting to note that NDRG1 is a known tumor suppressor that has been reported to be associated with stress and the prevalence of hypoxic conditions in tumors. Increased expression of NDRG1 is associated with poor patient outcomes which might be the case for meningiomas as well (32, 33).

### Components of Integrin and PI3K-Akt Pathways That Are Involved in Meningioma Pathobiology

Our study points out the prominent involvement of several components of the Focal Adhesion and Integrin pathway in meningiomas which has also been reported by other studies (34, 35). We found higher levels of FMNL2, ITGAM, and MRC2 among several other components mapping to the Focal adhesion and PI3K-Akt pathway in our patient cohort. While several candidates of the Focal Adhesion family have been associated with meningiomas via transcriptomic and proteomics studies; it is to be noted that most Integrins by themselves do not possess enzymatic activity (36). ILK, a 59 kDa protein kinase has emerged as an interesting player in mediating the signaling cascades through its interaction with members of the Integrin family as well as engaging several downstream effectors including AKT, GSK3, mTOR (37).

### Scope of ILK Inhibition as an Adjuvant Therapy

A study employing the inhibition of ILK showed that this affects cell growth in meningiomas by hindering the interaction of ILK with downstream components of PI3K-AKT (16). To determine the influence on inhibition of the ILK, one of the key components that regulate the three perturbed pathways namely Integrin, PI3K-Akt, and NF-κB, we have used the ILK inhibitor Cpd22 (Merck Millipore) on both meningioma patient-derived primary cell line as well as Ben-Men1. Inhibiting the activity of ILK affected the levels of key proteins known to be key players in meningioma pathobiology namely EIF4G1, CSNK2A1, and several others. At the transcriptomic level we observed the downregulation of EZR, GSN, IQGAP1, IQGAP2, and the several other Cytoskeletal components; AKT1, EIF4G1, ILK, and NFKB1 associated with PI3K-Akt and NF-κB. These findings need to be extrapolated in animal models for further substantiation of these observations. Overall, the perturbations observed using Cpd22 indicate that ILK inhibition has the potential to be used as an adjuvant therapy especially in cases wherein the tumor location makes complete resection difficult as well as for cases of multiple incidences of recurrence.

### Designing SRM Assay to Validate Clinically Relevant Protein Markers From Meningioma Patients

In a recent study by Diamandis et al., a global proteomic analysis of meningioma patient cohort of nearly 60 patient FFPE blocks were analyzed and many of the biomarkers that were validated in our current study namely SELENBP1, NDRG1, and MAPK3 were found aberrated in the FFPE samples indicating their true potential as markers for meningiomas that can even be used in clinics (10). Hence, for the first time SRM assay using a “patient-derived spectral library” was employed to monitor several potential meningioma biomarkers. Key candidates of the Focal adhesion pathway like ILK, EZR, MAP4, and VIM was also monitored from a larger patient cohort. The targeted proteomics approach enabled monitoring the levels of the several putative markers namely CLIC1, ES8L2, AHNAK many of which are receptors, and kinases and are difficult to be characterized using conventional approaches. Furthermore, using both label-free based proteomics as well as targeted proteomics we were able to cross-validate many of the proteins namely ANXA2, AHNAK, and CKAP4 that were identified in our earlier study (9).

## Conclusion

Combining global and targeted patient-derived data is a novel approach and it has the potential to provide cues regarding the meningioma pathobiology which can be useful in longitudinal studies for monitoring the patient prognosis. While inhibitory studies against FAK, mTOR, and Akt components in meningioma patients are ongoing (38); our study provides mechanistic insights on how components of Cytoskeletal regulators, PI3K-Akt, mTOR, and NFκB can be targeted using a single molecule namely the Cpd22 which was able to downplay these components in meningioma primaries. We also lay the foundation for the use of targeted proteomics for validation of key proteins directly from the patient cohort which if integrated with existing modalities of diagnosis and treatment can aid in enhanced patient management.

## Data Availability Statement

The mass spectrometry proteomics datasets have been deposited to the ProteomeXchange Consortium via the PRIDE partner repository with the dataset identifier PXD014852, PXD014853, PXD014855, and PXD014823.

## Ethics Statement

The studies involving human participants were reviewed and approved by TMH and IITB IEC. The patients/participants provided their written informed consent to participate in this study.

## Author Contributions

SM carried out all experiments and compiled the manuscript. SS, AMo, SE, and SM conceived the study and secured funding. AMo, PS, and SE performed the clinical, surgical, and pathological examination of the patients. DB performed GSEA analysis. GR and PJ performed machine learning-based analysis. SM performed SRM assay experiments. RG was involved in optimizations. NS facilitated MG cell line assays. RT^2^ PCR assay was performed by JS and QZ. All authors contributed to the article and approved the submitted version.

## Conflict of Interest

The authors declare that the research was conducted in the absence of any commercial or financial relationships that could be construed as a potential conflict of interest.
