# Supplementary material for: Comprehending Meningioma Signaling Cascades Using Multipronged Proteomics Approaches & Targeted Validation of Potential Markers
Source: Front Oncol. 2020 Aug 26;10:1600. doi: 10.3389/fonc.2020.01600 (PMC7482667; doi:10.3389/fonc.2020.01600)
Supplement: Supplementary file 1 [file Table_1.docx]

Supplementary Data 1

**Patients Details (Label Free Global Proteomic Analysis)**

| **S.No.** | **Patient ID** | **Diagnosis** | **Grade** | **Age** | **Sex** | **Radiology** |
| --- | --- | --- | --- | --- | --- | --- |
| 1 | MN LFQ1 | Invasive Atypical Meningioma | II | 64 | F | Supratentorial |
| 2 | MN LFQ2 | Atypical Meningioma | II | 53 | M | Supratentorial |
| 3 | MN LFQ3 | Meningioma | I | 45 | F | Supratentorial |
| 4 | MN LFQ4 | Meningothelial tumor With Atypical Feature | II | 31 | M | Supratentorial |
| 5 | MN LFQ5 | Atypical Meningioma | II | 35 | F | Supratentorial |
| 6 | MN LFQ6 | Meningioma | I | 44 | F | Supratentorial |
| 7 | MN LFQ7 | Atypical Menigothelial Meningioma | II | 46 | F | Supratentorial |
| 8 | MN LFQ8 | Meningothelial Meningioma | I | 31 | M | Skull base |
| 9 | MN LFQ9 | Transitional Meningioma | I | 42 | F | Skullbase |
| 10 | MN LFQ10 | Atypical Meningioma | II | 51 | F | Supratentorial |
| 11 | MN LFQ11 | Transitional Meningioma | II | 50 | F | Supratentorial |
| 12 | MN LFQ12 | Atypical Meningioma | II | 45 | F | Supratentorial |
| 13 | MN LFQ13 | Transitional Meningioma | I | 40 | F | Skullbase |
| 14 | MN LFQ14 | Transitional Meningioma | II | 32 | F | Skullbase |
| 15 | MN LFQ15 | Atypical Meningioma | II | 62 | F | Supratentorial |
| 16 | MN LFQ16 | Meningioma, | I | 54 | F | Skullbase |
| 17 | MN LFQ17 | Transitional Meningioma | I | 57 | F | Supratentorial |
| 18 | MN LFQ18 | Fibroblastic Meningioma | I | 37 | F | Skullbase |
| 19 | MN LFQ19 | Transitional Meningioma | I | 66 | F | Supratentorial |
| 20 | MN LFQ20 | Meningothelial Meningioma | II | 54 | M | Skullbase |
| 21 | MN LFQ21 | Transitional Meningioma | I | 39 | M | Supratentorial |

**Control**

| **S.No.** | **Patient ID** | **Anatomical Area** | **Age** | **Sex** |
| --- | --- | --- | --- | --- |
| 1 | T387 | Dura mater | 22 | M |
| 2 | T388 | Dura mater | 28 | M |
| 3 | T390 | Dura mater | 45 | M |
| 4 | T391 | Dura matter | 36 | M |
| 5 | B292 | Dura mater | 86 | M |
| 6 | B291 | Arachnoid | 6 Months | F |
| 7 | B293 | Arachnoid | 88 | M |
| 8 | B296 | Arachnoid | 85 | M |

**Phosphoproteomics Analysis of Meningioma Patient Cohort**

| **S.No.** | **Patient ID** | **Diagnosis** | **Grade** | **Age** | **Sex** |
| --- | --- | --- | --- | --- | --- |
| 1 | MN P1 | Invasive Atypical Meningioma | II | 64 | F |
| 2 | MN P2 | Atypical Meningioma | II | 53 | M |
| 3 | MN P3 | Meningioma | I | 45 | F |
| 4 | MN P4 | Meningothelial Tumour With Atypical Feature | II | 31 | M |
| 5 | MN P5 | Atypical Meningioma | II | 35 | F |
| 6 | MN P6 | Meningioma | I | 44 | F |
| 7 | MN P7 | Atypical Menigothelial Meningioma | II | 46 | F |
| 8 | MN P8 | Meningothelial Meningioma | I | 31 | M |
| 9 | MN P9 | Transitional Meningioma | I | 42 | F |
| 10 | MN P10 | Transitional Meningioma | II | 50 | F |
| 11 | MN P11 | Atypical Meningioma | II | 45 | F |
| 12 | MN P12 | Transitional Meningioma | I | 40 | F |
| 13 | MN P13 | Transitional Meningioma | II | 32 | F |
| 14 | MN P14 | Atypical Meningioma | II | 62 | F |
| 15 | MN P15 | Meningioma, | I | 54 | F |
| 16 | MN P16 | Transitional Meningioma | I | 52 | F |
| 17 | MN P17 | Atypical Meningioma | II | 50 | F |
| 18 | MN P18 | Atypical Meningioma | II | 32 | F |

**IP-MS POOL**

| **S.No.** | **Patient ID** | **Diagnosis** | **Grade** | **Age** | **Sex** |
| --- | --- | --- | --- | --- | --- |
| 1 | MN-IP1 | Transitional Meningioma | I | 31 | F |
| 2 | MN-IP2 | Meningothelial Meningioma | I | 54 | F |
| 3 | MN-IP3 | Transitional Meningioma | I | 39 | M |

**SRM Assay in Meningioma Patient Cohort**

| Sample ID | RADIOLOGICAL SEGREGATION | GRADES | Age | Sex |
| --- | --- | --- | --- | --- |
| MN1 | skull base | II | 50 | M |
| MN2 | supratentorial | II | 46 | F |
| MN3 | supratentorial | I | 62 | F |
| MN4 | supratentorial | I | 49 | F |
| MN5 | supratentorial | I | 41 | F |
| MN6 | supratentorial | I | 57 | F |
| MN7 | skull base | I | 53 | M |
| MN8 | supratentorial | II | 64 | F |
| MN9 | skull base | II | 47 | M |
| MN10 | supratentorial | II | 53 | M |
| MN11 | skull base | I | 58 | F |
| MN12 | supratentorial | II | 58 | F |
| MN13 | supratentorial | I | 57 | F |
| MN14 | skull base | II | 33 | M |
| MN15 | skull base | I | 37 | F |
| MN16 | skull base | I | 28 | M |
| MN17 | supratentorial | II | 62 | F |
| MN18 | skull base | I | 46 | F |
| MN19 | skull base | I | 32 | F |
| MN20 | supratentorial | II | 50 | M |
| MN21 | supratentorial | II | 54 | F |
| MN22 | skull base | II | 57 | F |
| MN23 | supratentorial | II | 54 | F |
| MN24 | supratentorial | II | 67 | M |
| MN25 | supratentorial | II | 62 | F |
| MN26 | skull base | I | 60 | M |
| MN27 | skull base | I | 52 | F |
| MN28 | skull base | I | 54 | F |
| MN29 | supratentorial | NA | 62 | F |
| MN30 | skull base | I | 47 | F |
| MN31 | skull base | II | 32 | F |
| MN32 | skull base | II | 28 | F |
| MN33 | skull base | I | 60 | M |
| MN34 | skull base | II | 50 | M |
| MN35 | supratentorial | I | 69 | M |
| MN36 | skull base | I | 56 | M |
| MN37 | supratentorial | I | 66 | F |
| MN38 | supratentorial | I | 45 | M |
| MN39 | supratentorial | II | 55 | M |
| MN40 | skull base | II | 54 | M |
| MN41 | skull base | I | 37 | F |
| MN42 | supratentorial | II | 64 | F |
| MN43 | supratentorial | II | 53 | M |
| MN44 | skull base | I | 31 | M |
| MN45 | supratentorial | II | 62 | F |
| MN46 | supratentorial | I | 57 | F |
| MN47 | supratentorial | I | 39 | M |
| MN48 | supratentorial | I | 72 | M |
| MN49 | skull base | I | 53 | M |
| MN50 | supratentorial | I | 47 | F |
| MN51 | supratentorial | I | 52 | F |
| MN52 | supratentorial | III | 61 | M |
| MN53 | skull base | I | 63 | F |
| MN54 | skull base | I | 63 | F |
