# Supplementary material for: Comprehending Meningioma Signaling Cascades Using Multipronged Proteomics Approaches & Targeted Validation of Potential Markers
Source: Front Oncol. 2020 Aug 26;10:1600. doi: 10.3389/fonc.2020.01600 (PMC7482667; doi:10.3389/fonc.2020.01600)
Supplement: Supplementary file 10 [file Data_Sheet_1.docx]

**Supplementary Figures**

| **Title** | **Content** |
| --- | --- |
| **Supplementary Figure 1** | **Details of Proteome Discoverer® Parameters & Outcomes** |
| **Supplementary Figure 2** | **Alterations of the Cytoskeletal regulators upon treatment of Meningioma Primaries with Cpd22 (2.5uM)** |
| **Supplementary Figure 3** | **Alterations of the PI3K-Akt components upon treatment of Meningioma Primaries with Cpd22 (2.5uM)** |
| **Supplementary Figure 4** | **Cell Images: Treated vs Untreated cell lines** |
| **Supplementary Figure 5** | **SRM Analysis outcomes in various grades of meningioma and non tumor controls** |
| **Supplementary Figure 6** | **Perturbations in Integrin components in meningiomas** |


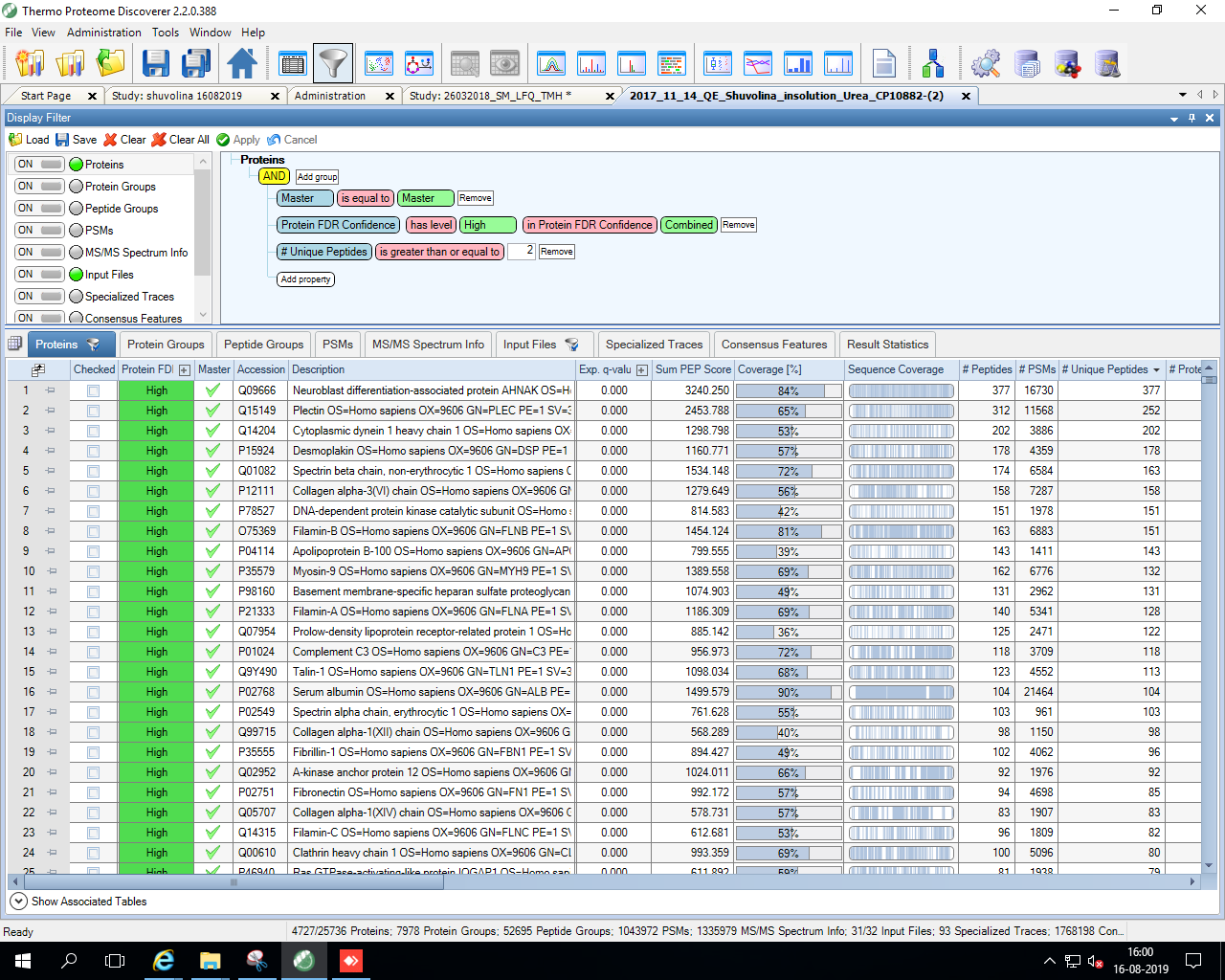

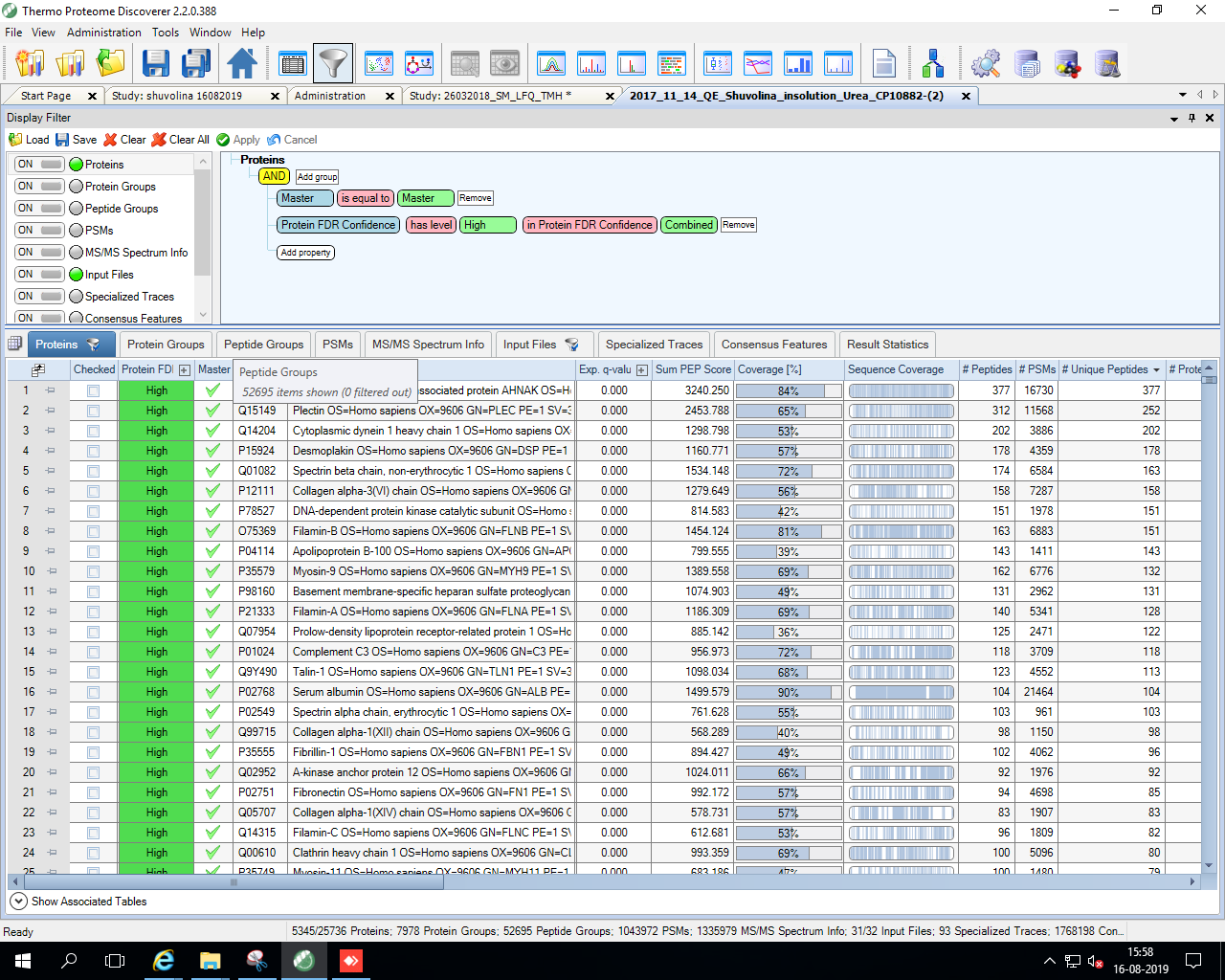


**Supplementary Figure 1: Details of Proteome Discoverer® Parameters & Outcomes**


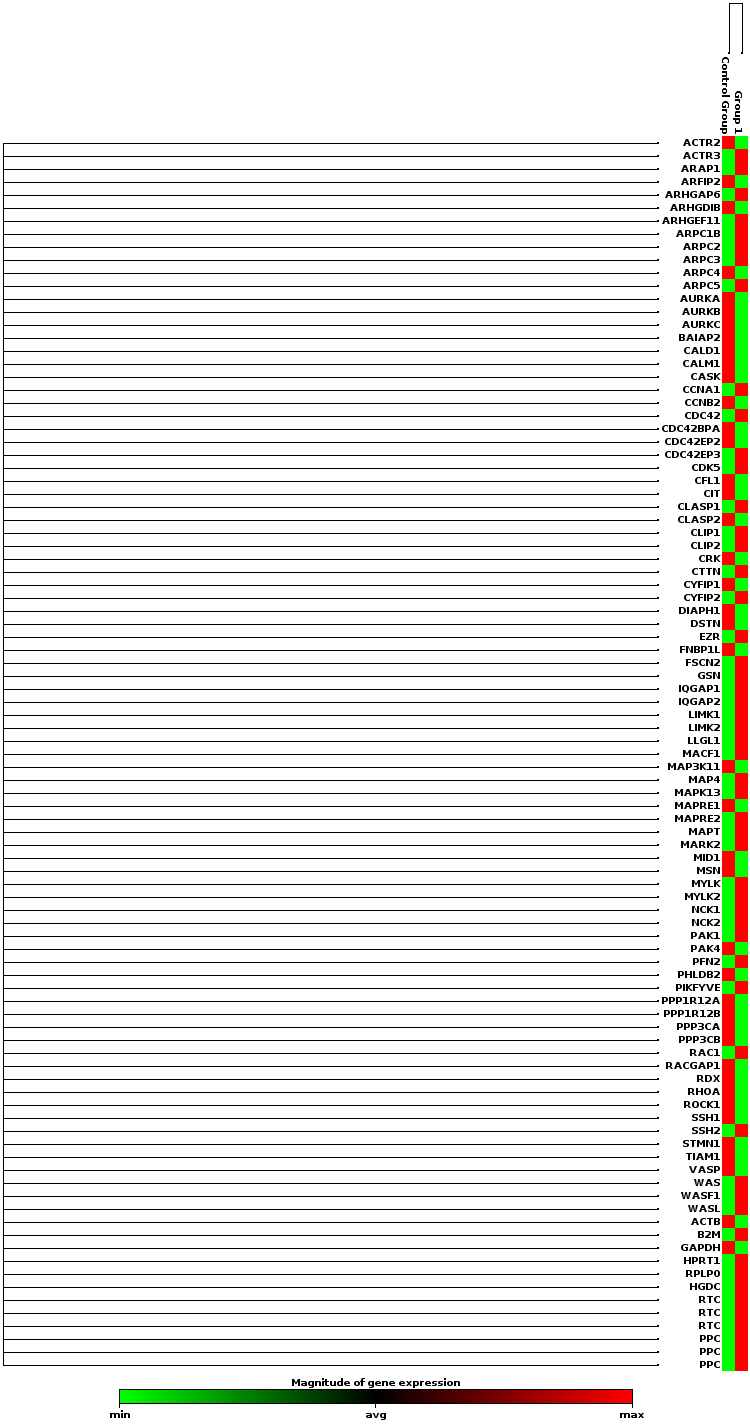


**Supplementary Figure 2: Alterations of the Cytoskeletal regulators upon treatment of Meningioma Primaries with Cpd22 (2.5uM)**


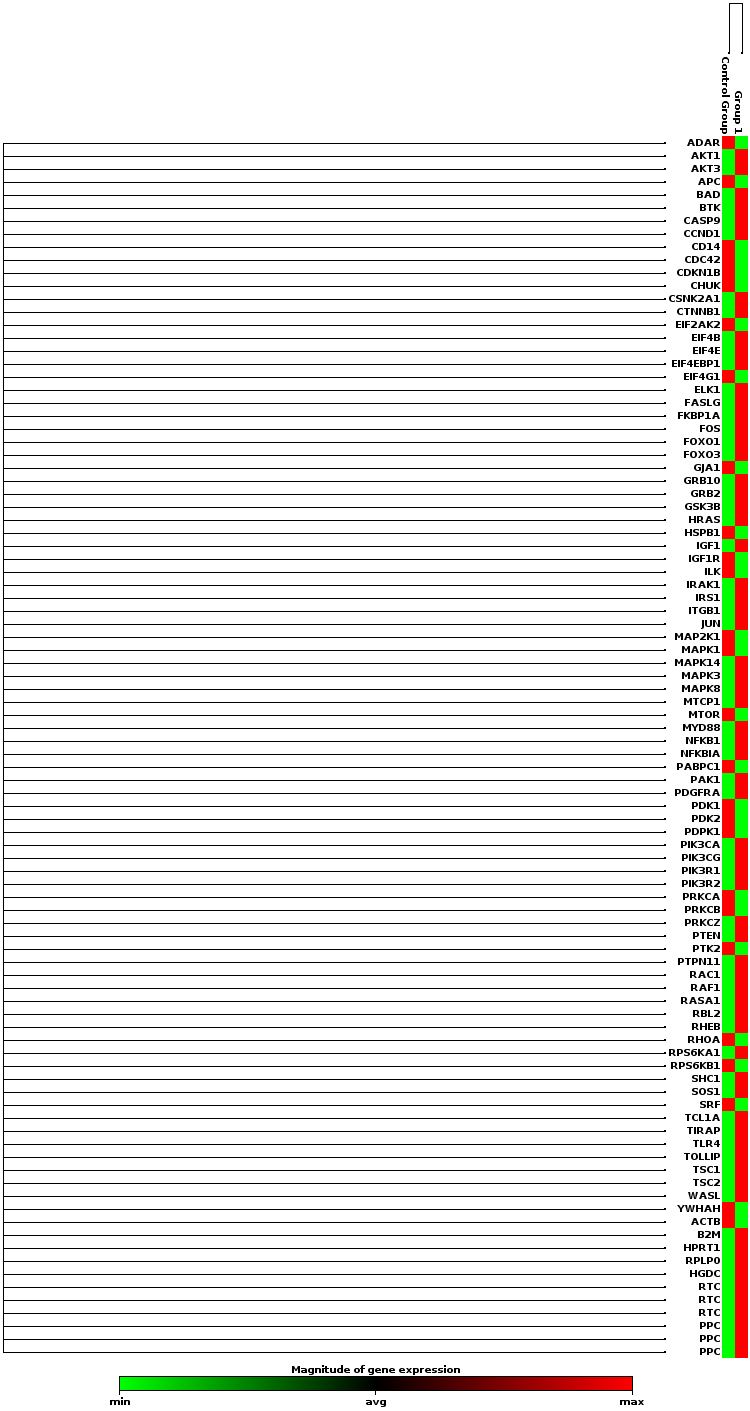


**
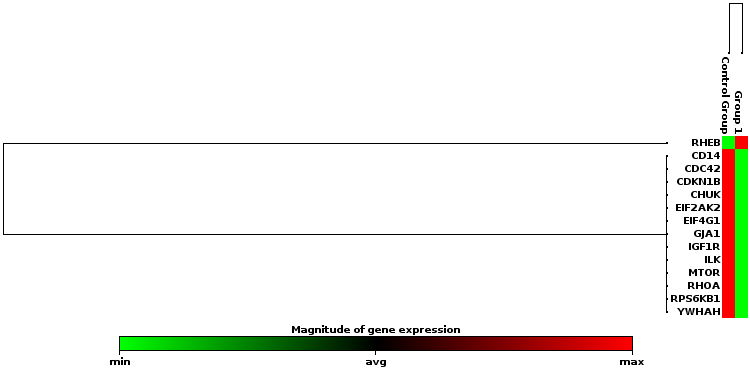
**

**Supplementary Figure 3: Alterations of the PI3-Akt components upon treatment of Meningioma Primaries with Cpd22 (2.5uM)**

**


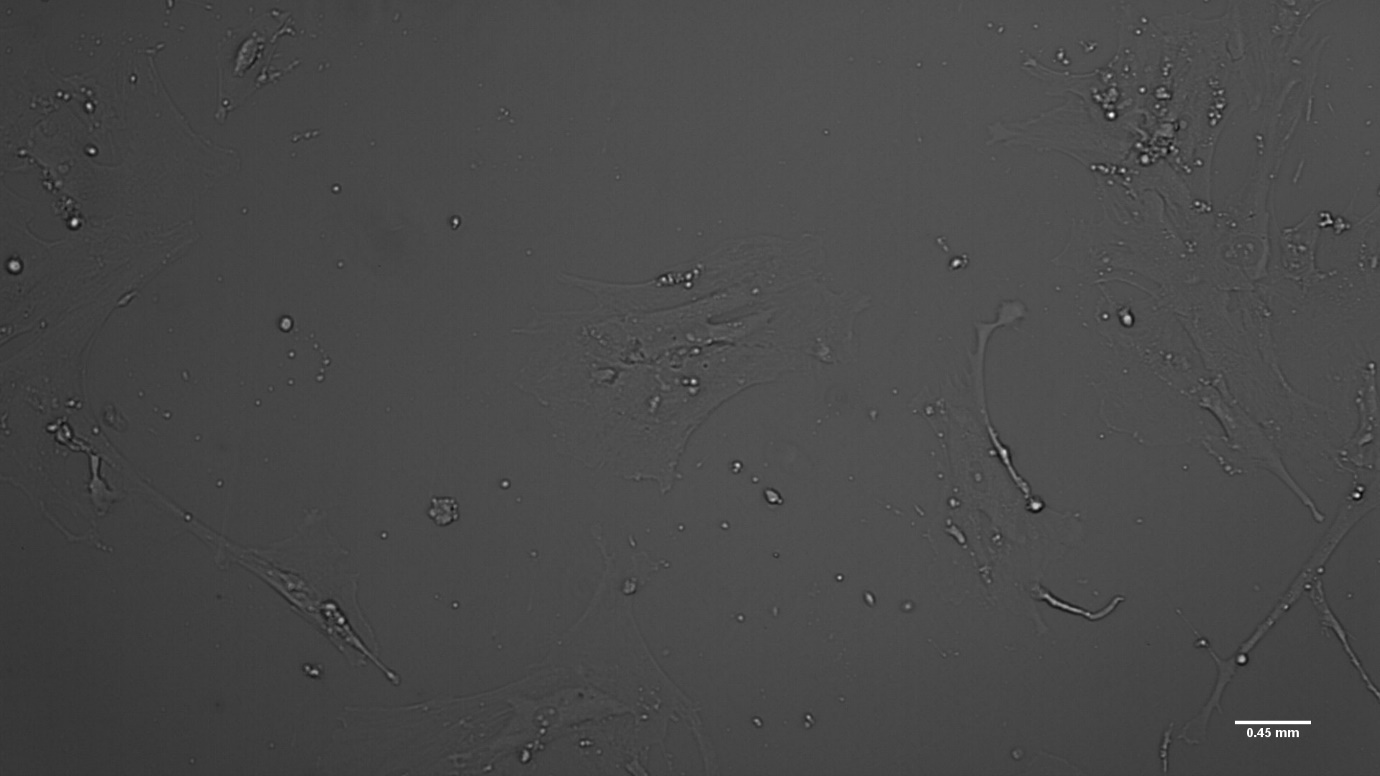
**

A. Untreated Meningioma Primaries (No Drug)

B. Treated Meningioma Primaries (2.5uM Cpd22 for 24 Hrs.)










**Supplementary Figure 4:**

**Cell Images: Treated vs Untreated cell lines as seen under light microscope post treatment with ILK inhibitor Cpd22**


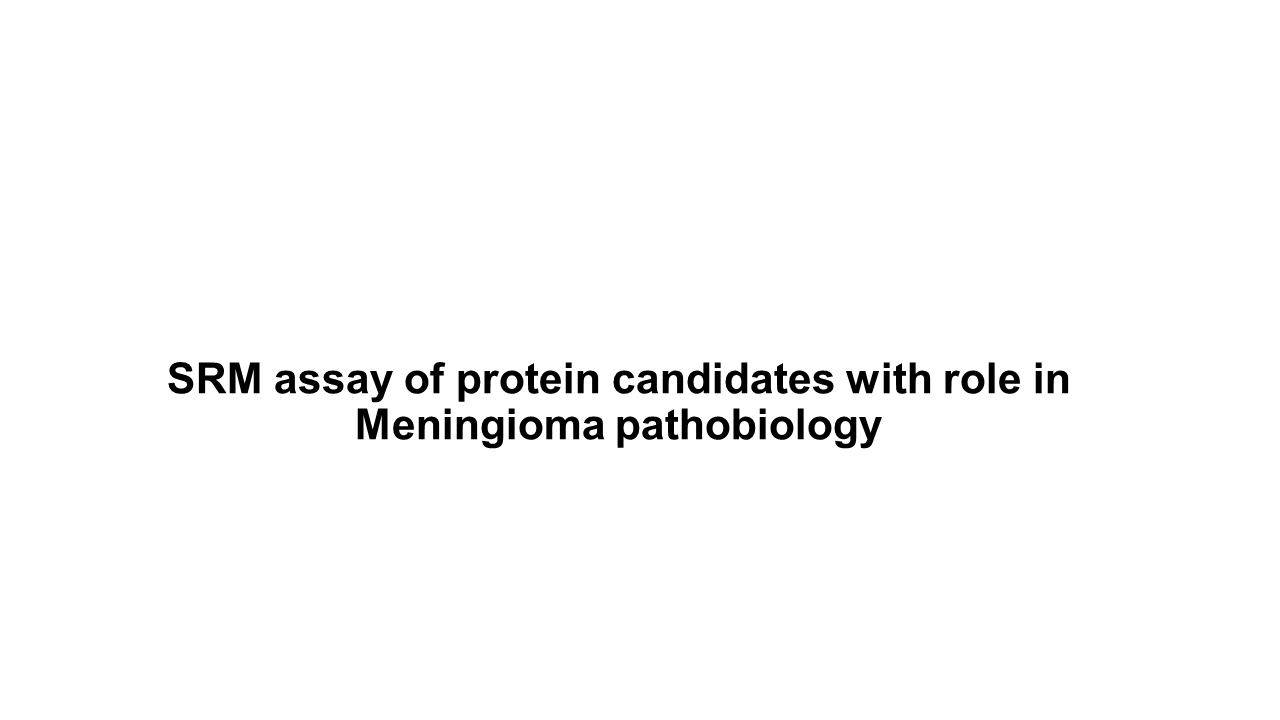

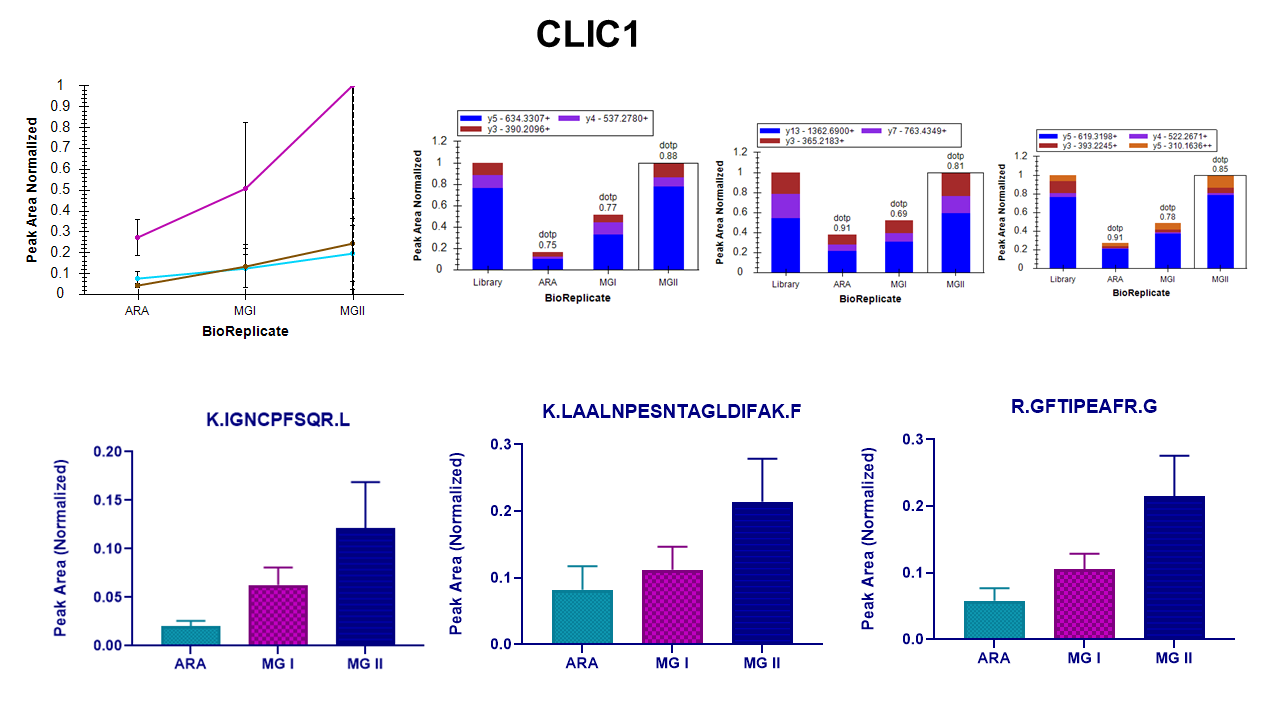

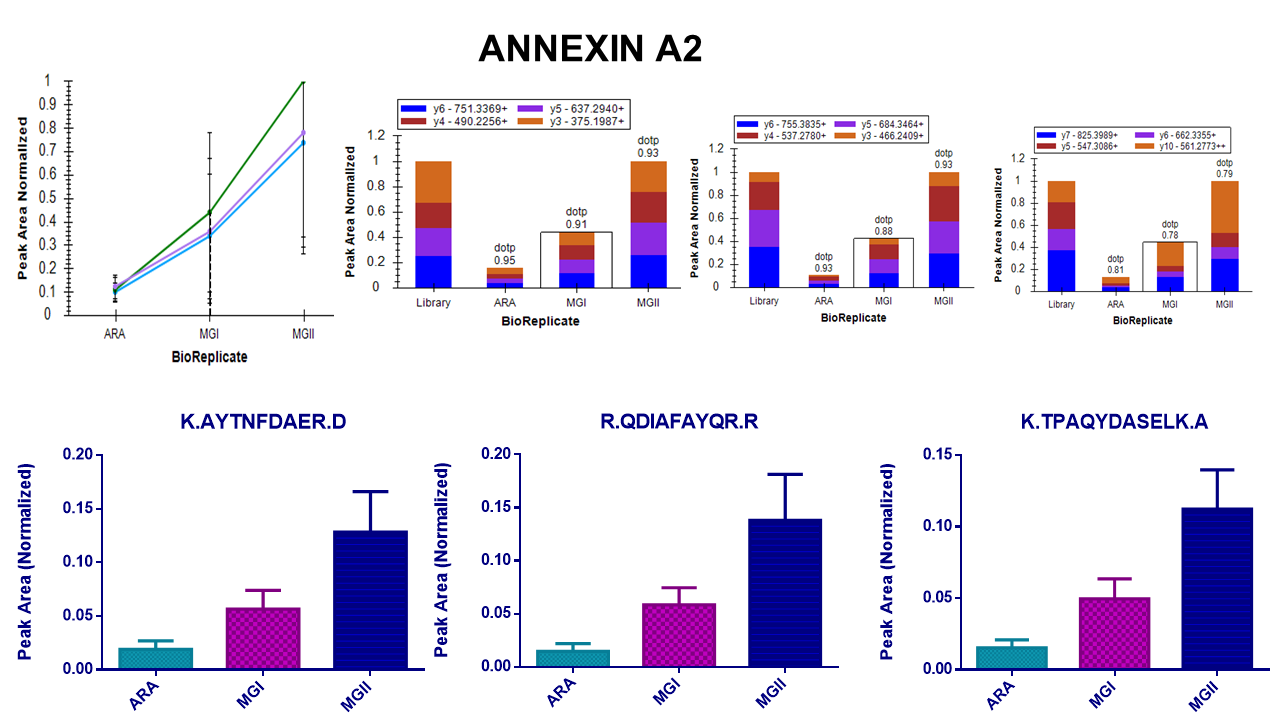

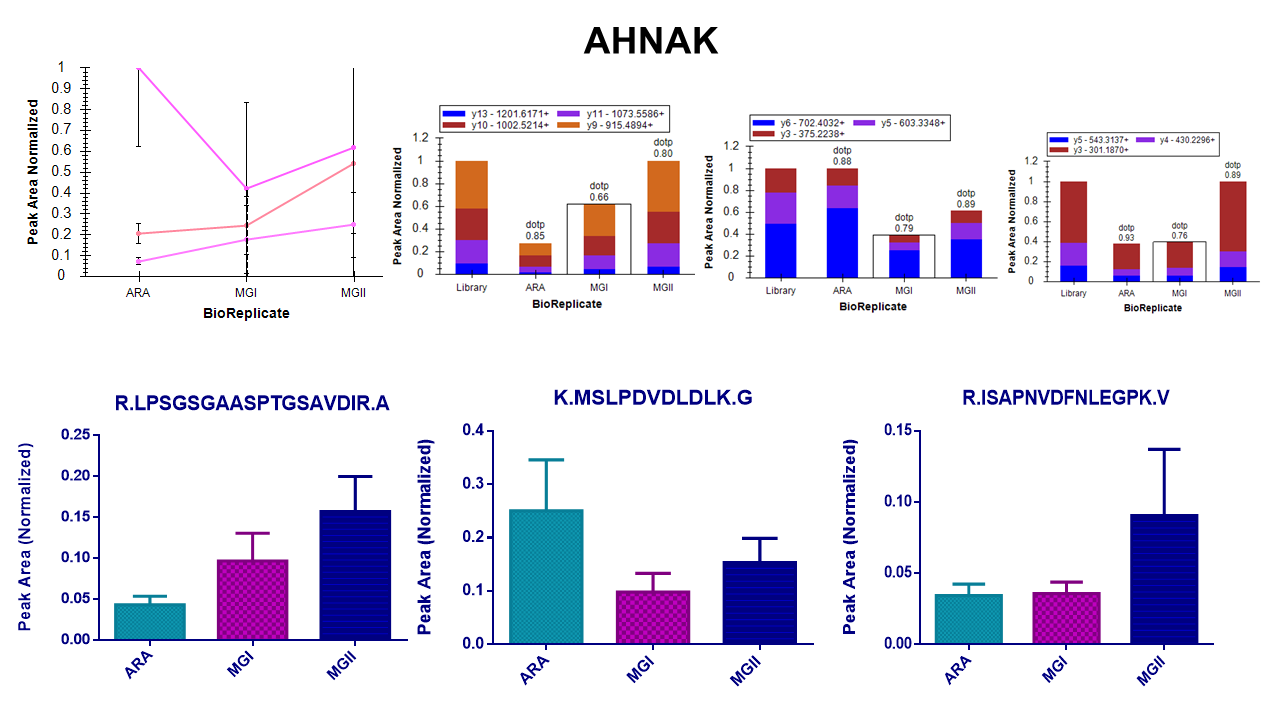

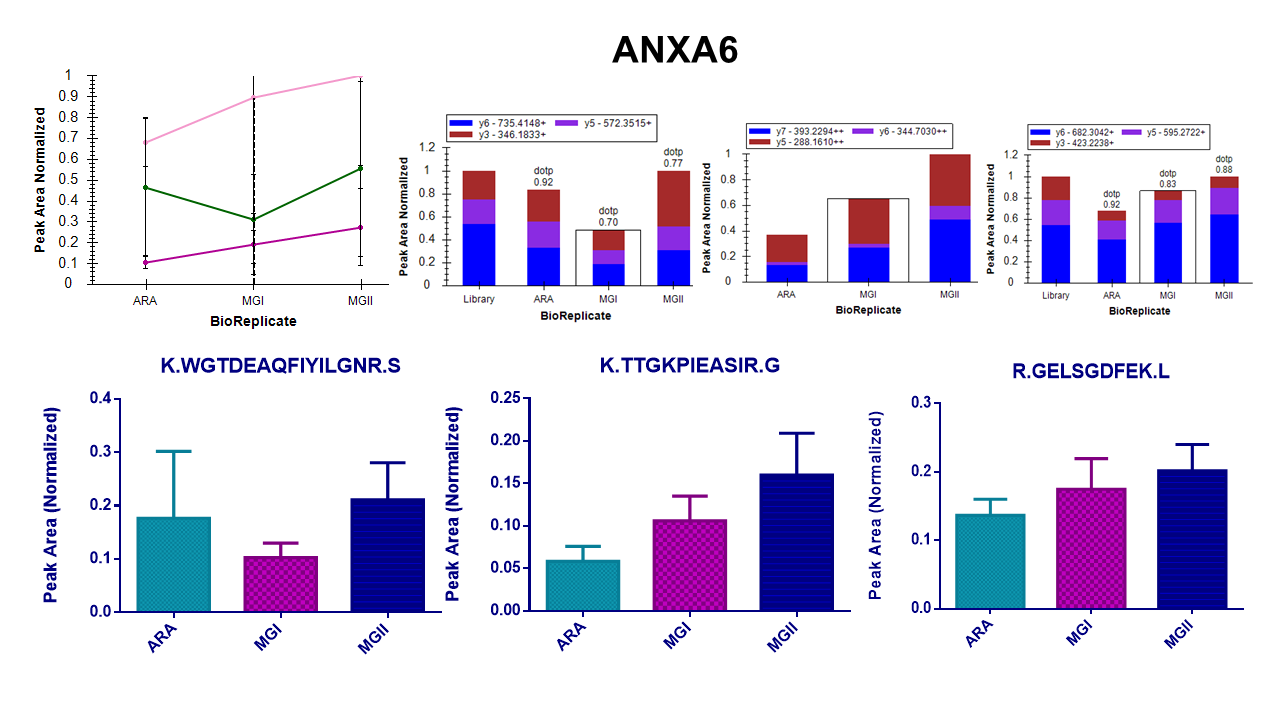

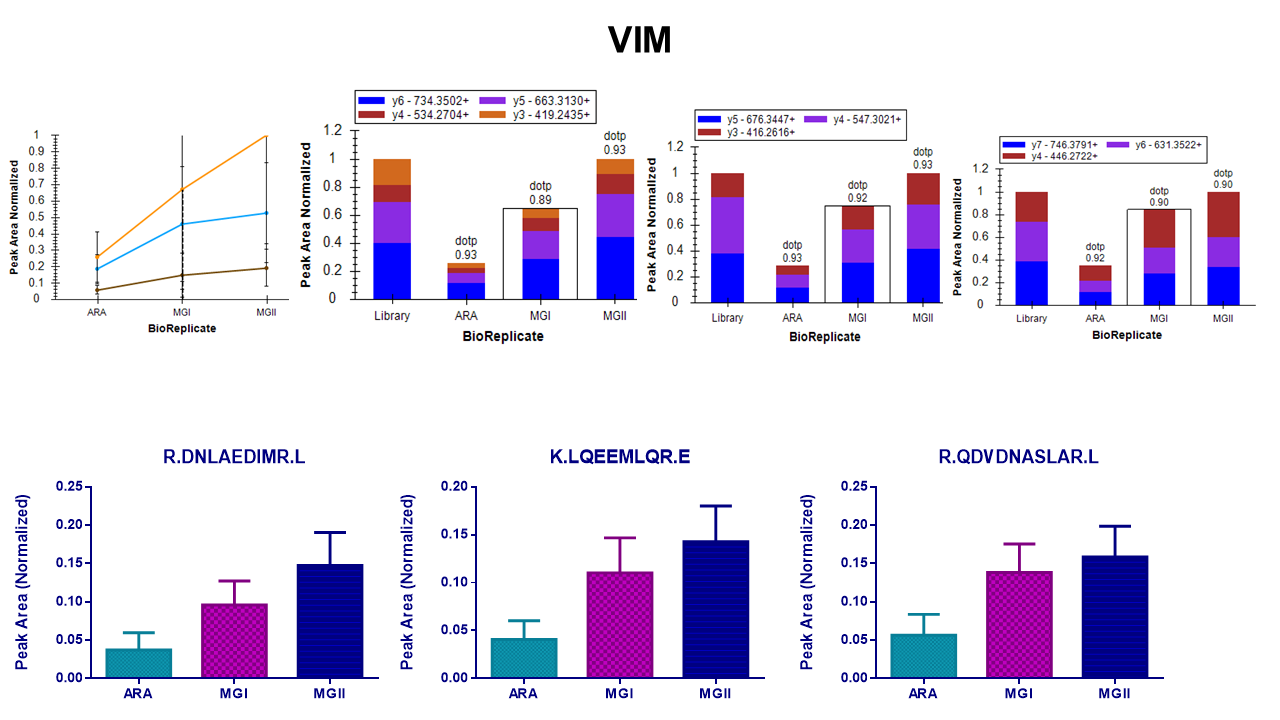

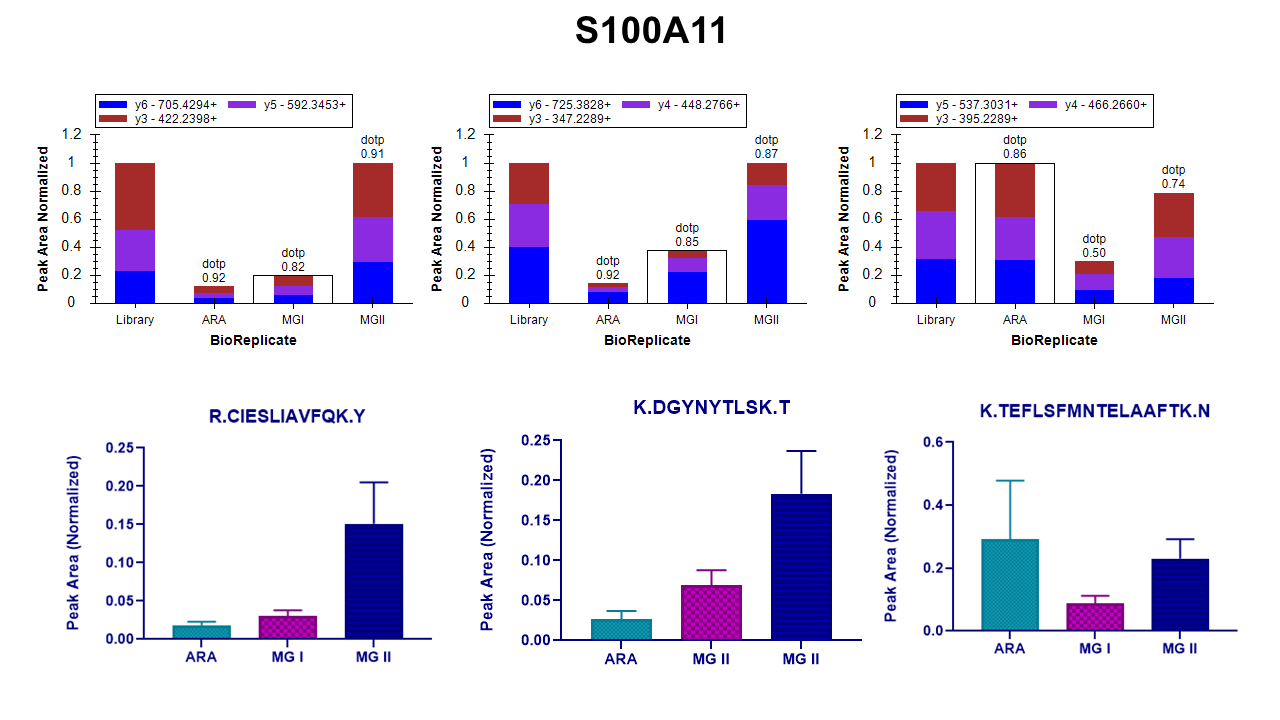

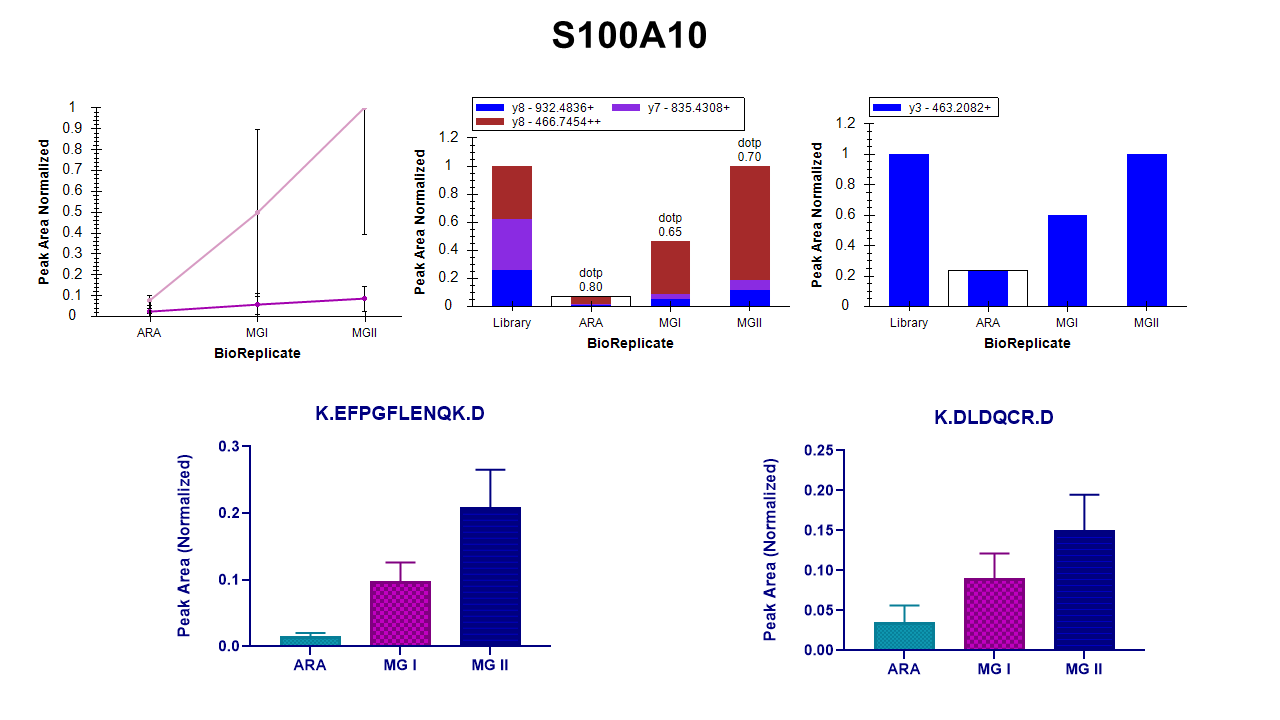

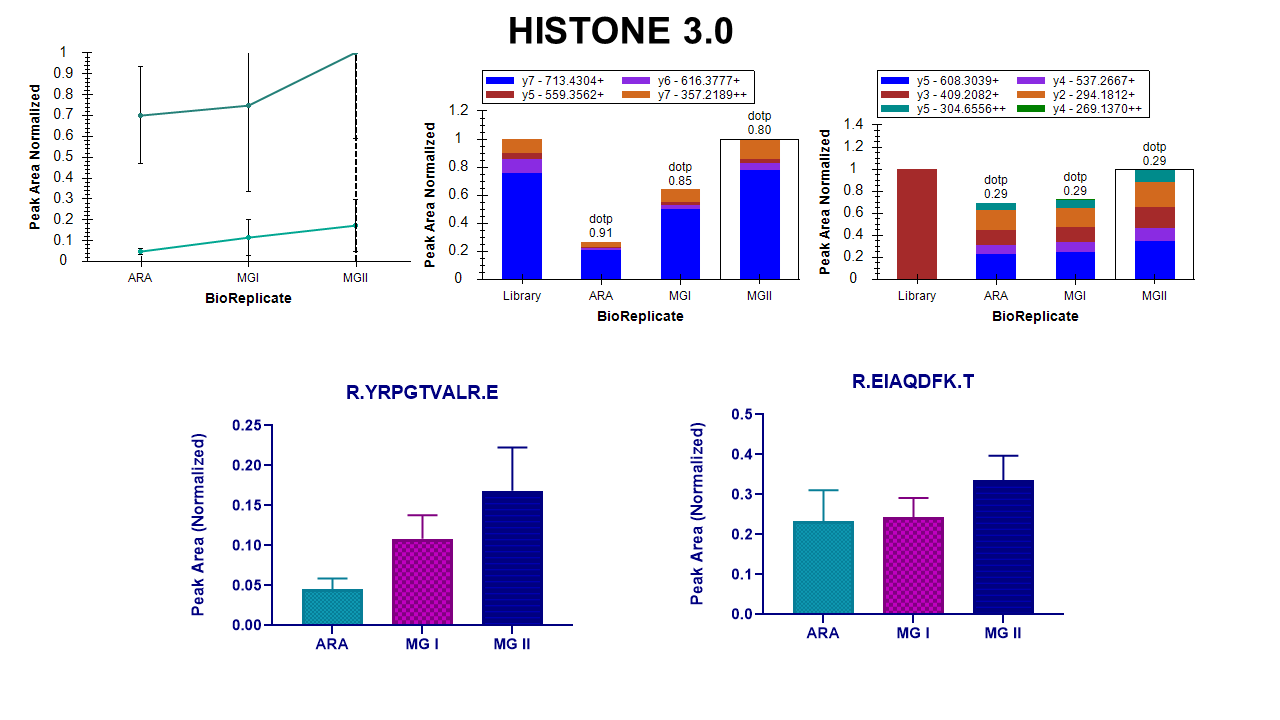

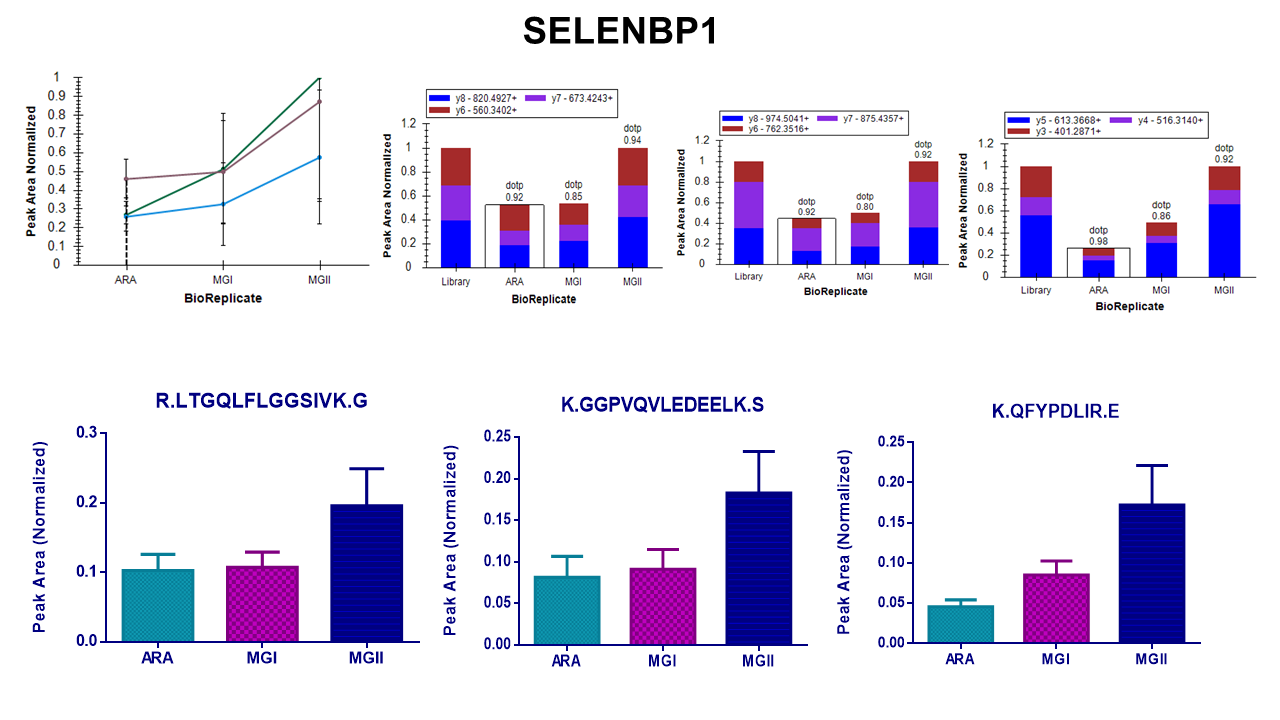

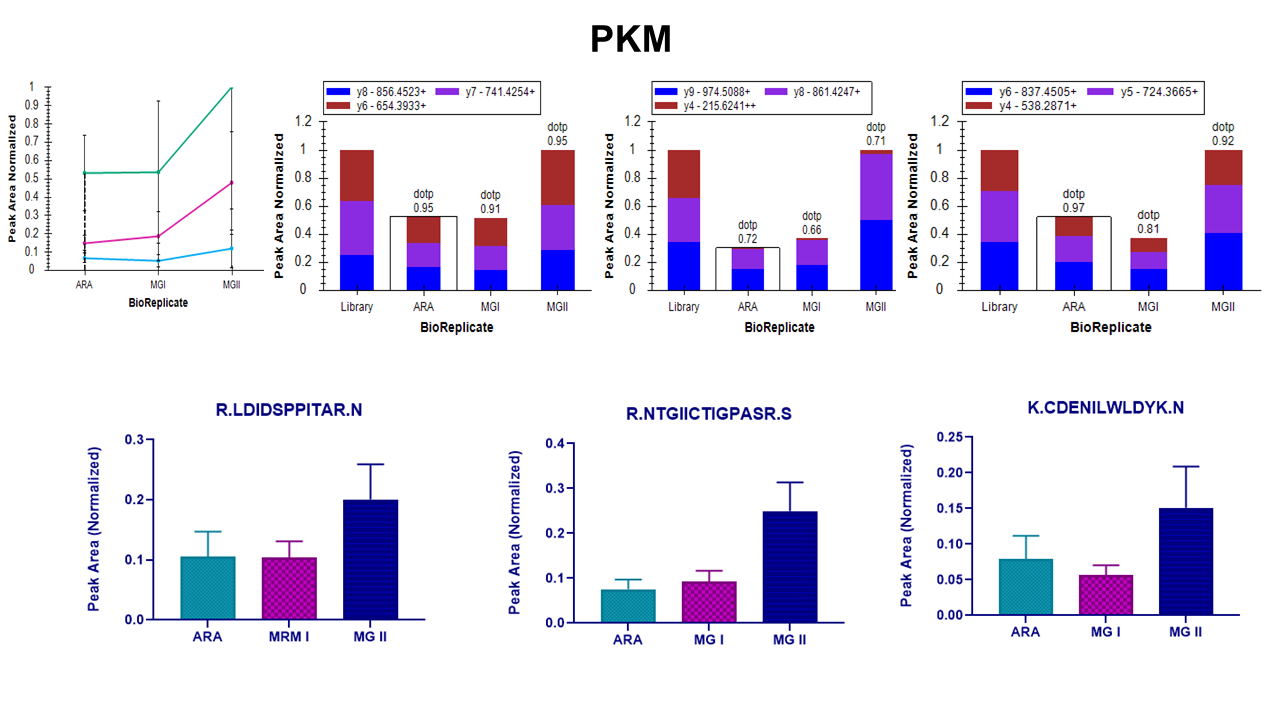

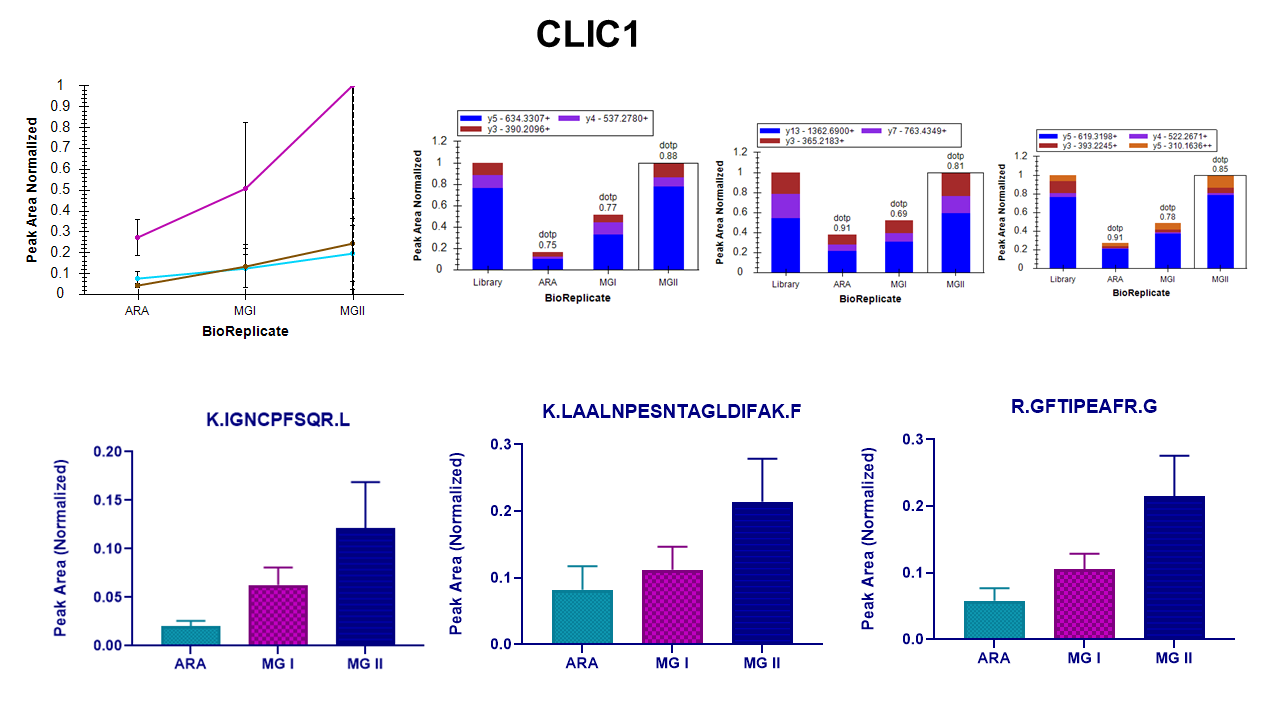

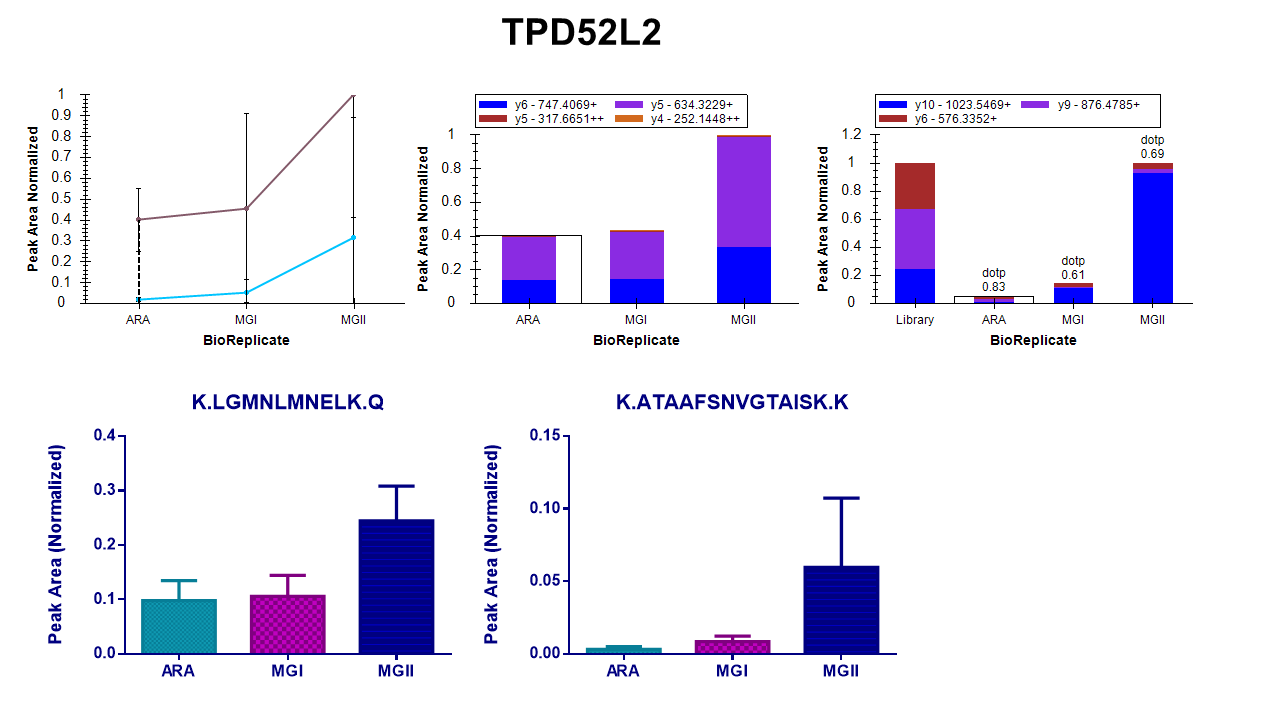

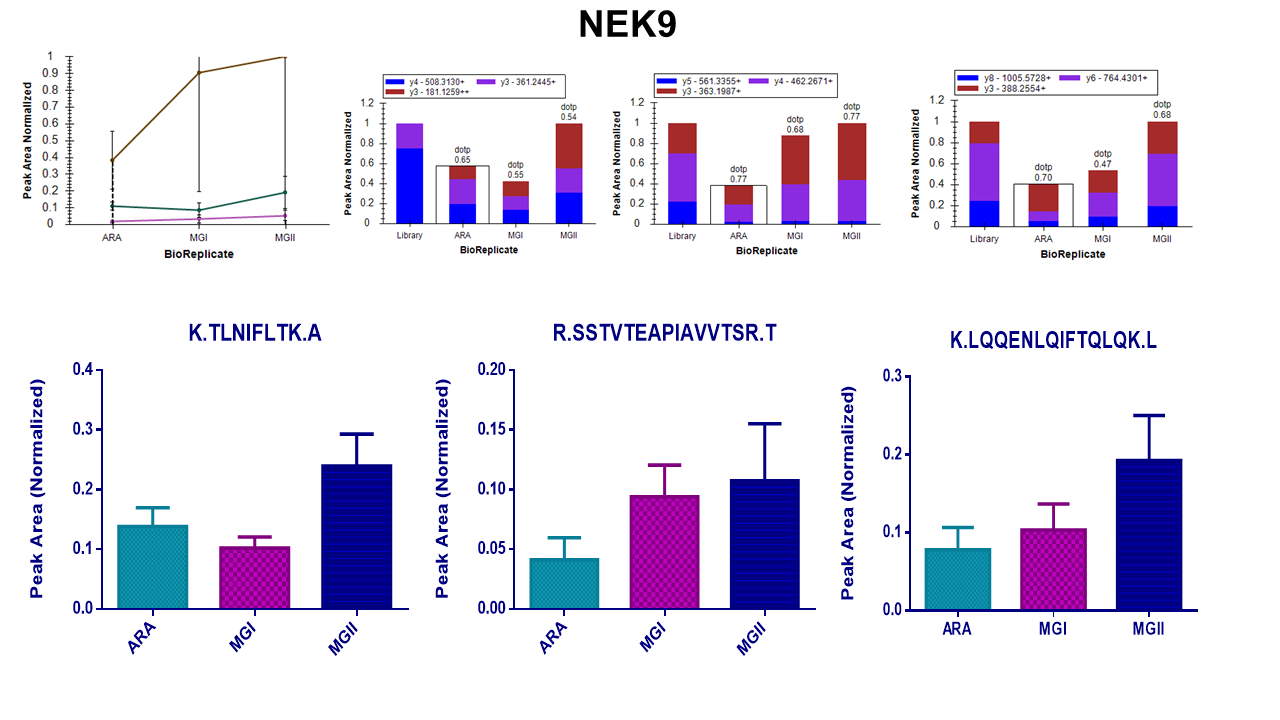

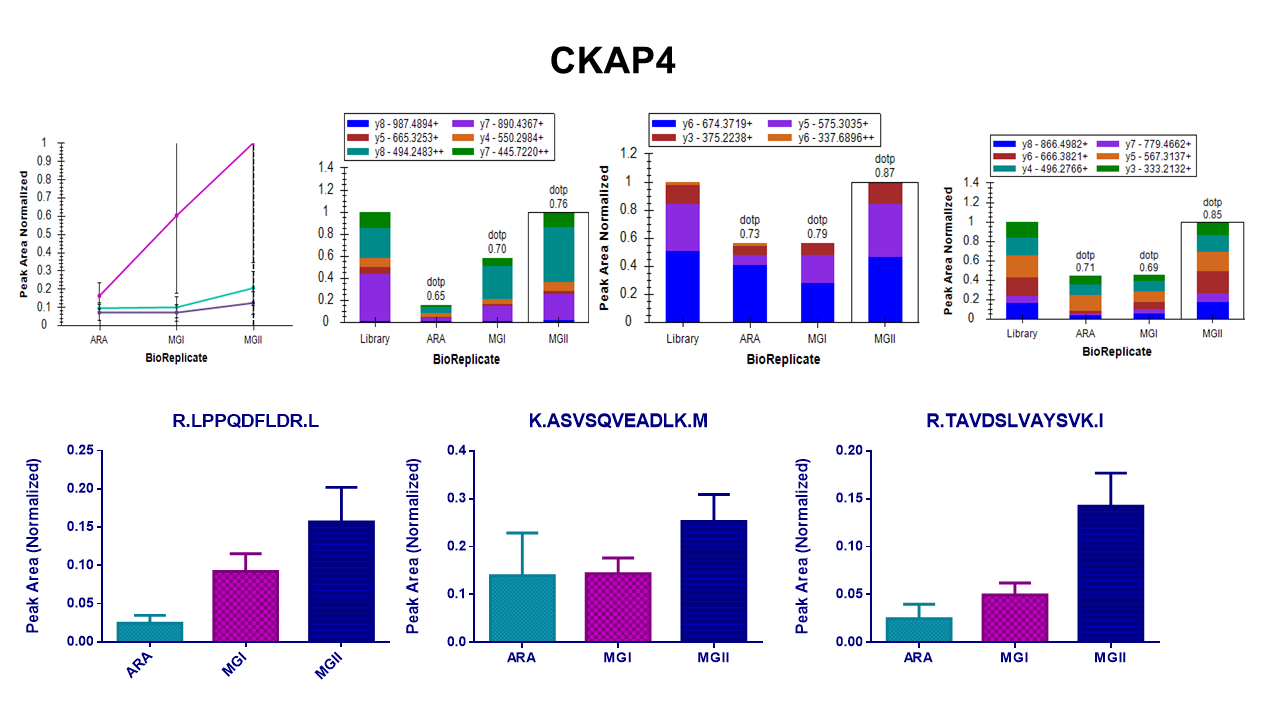

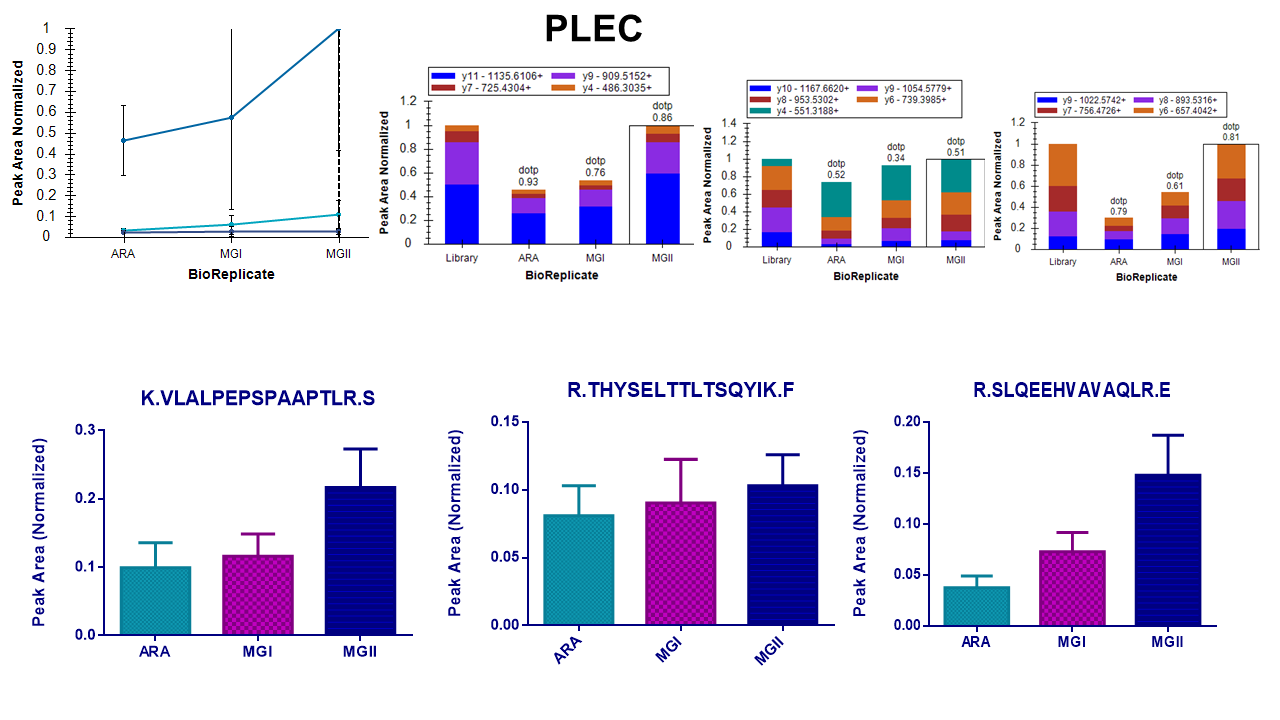

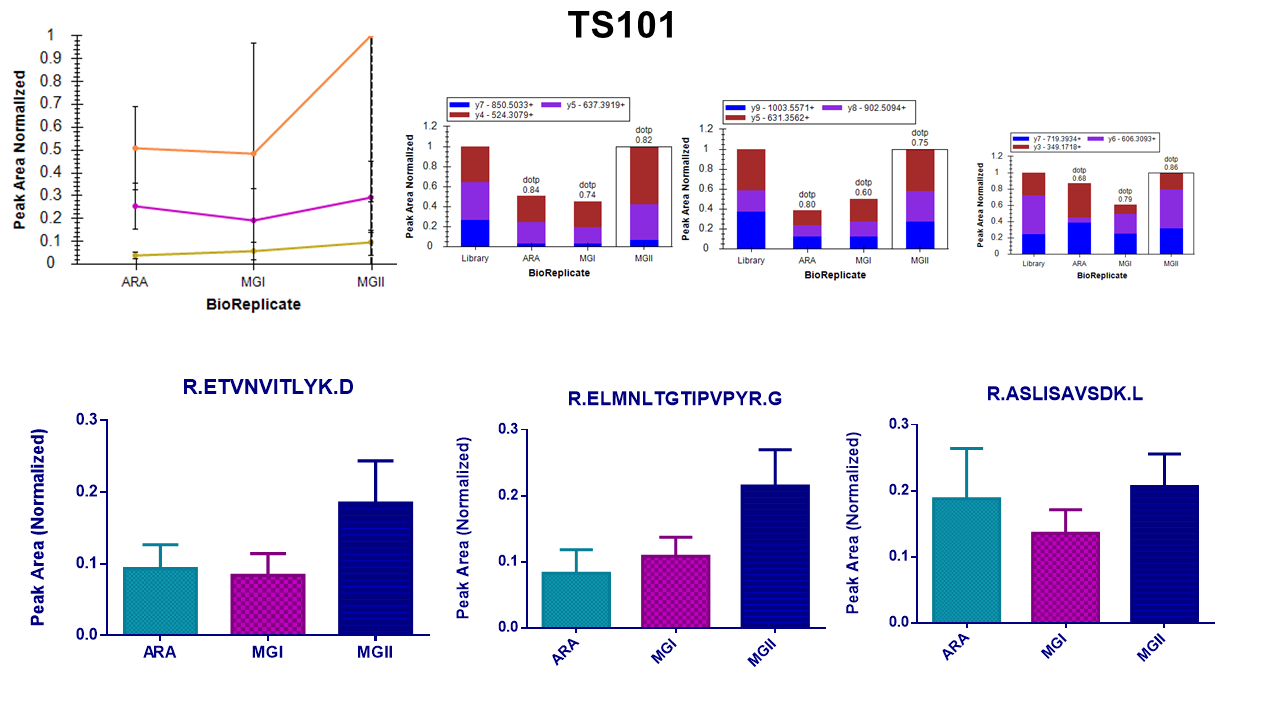

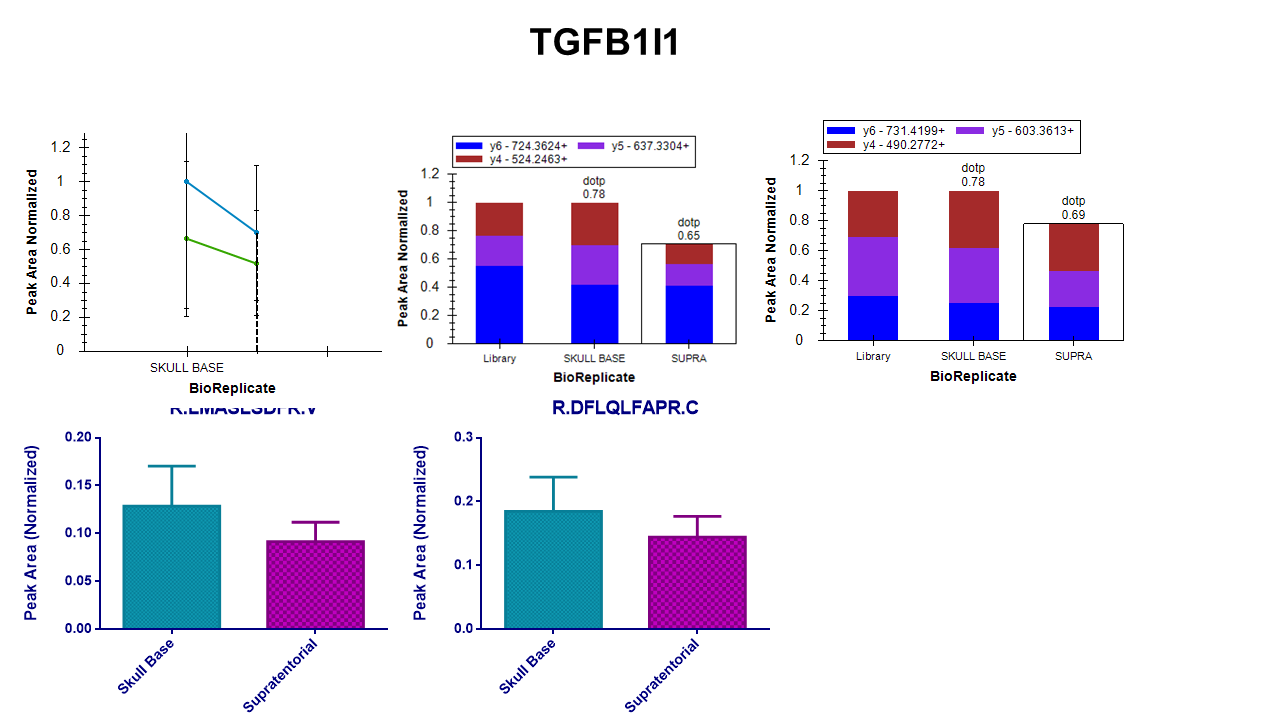

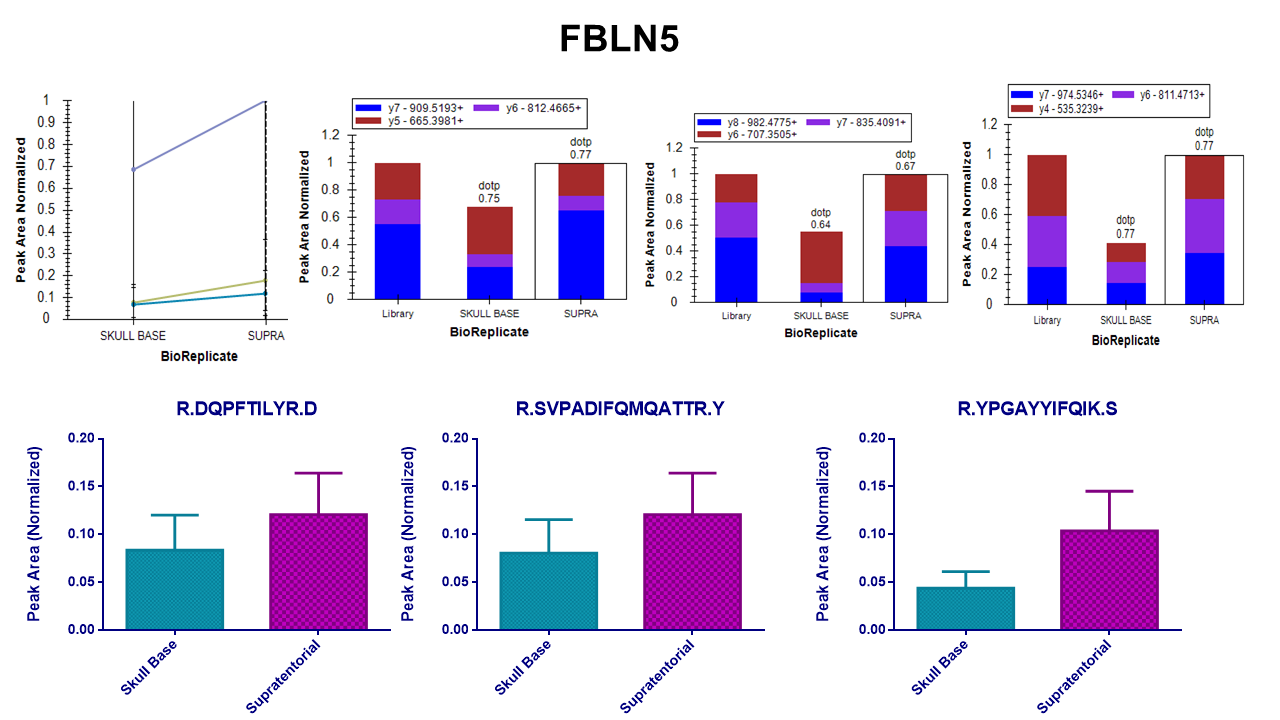

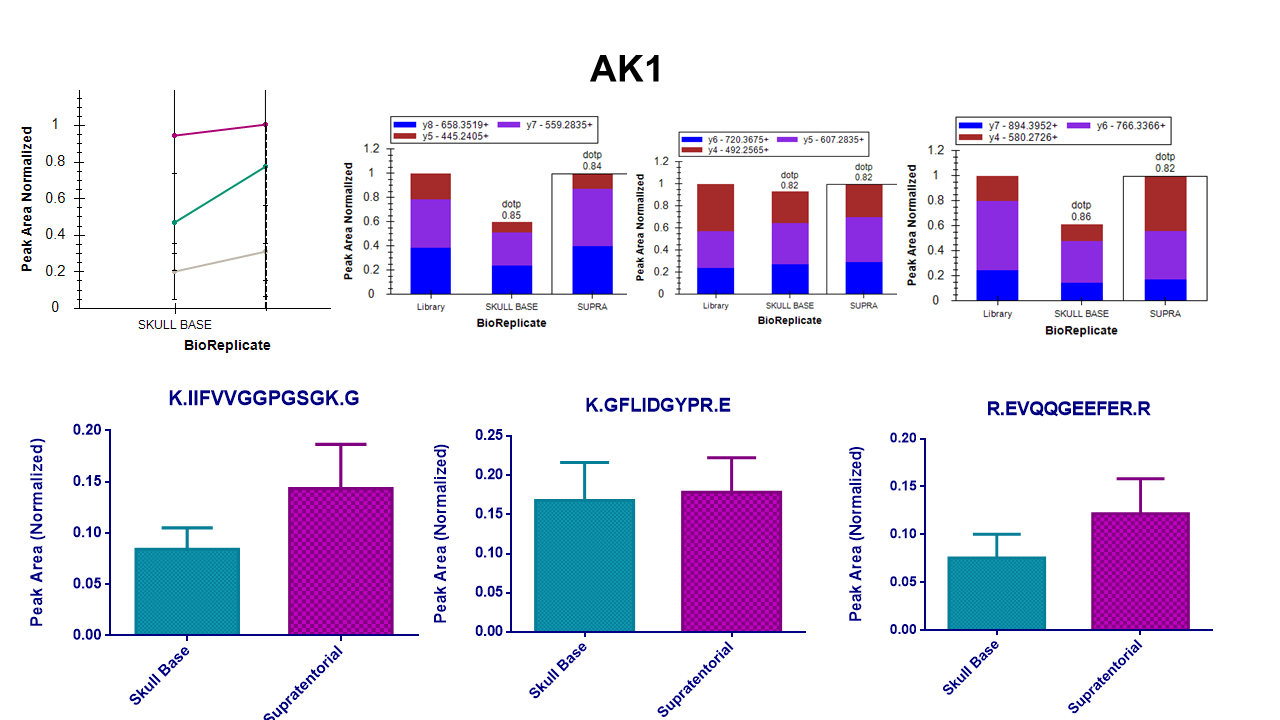

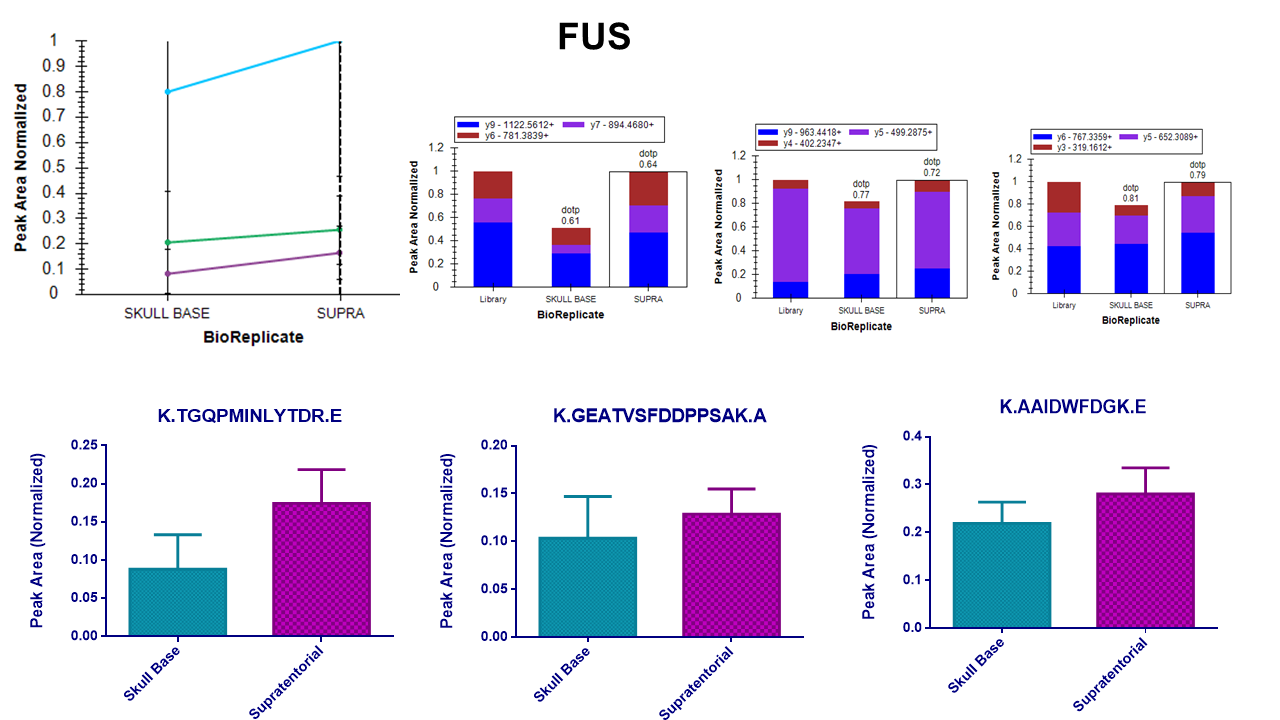

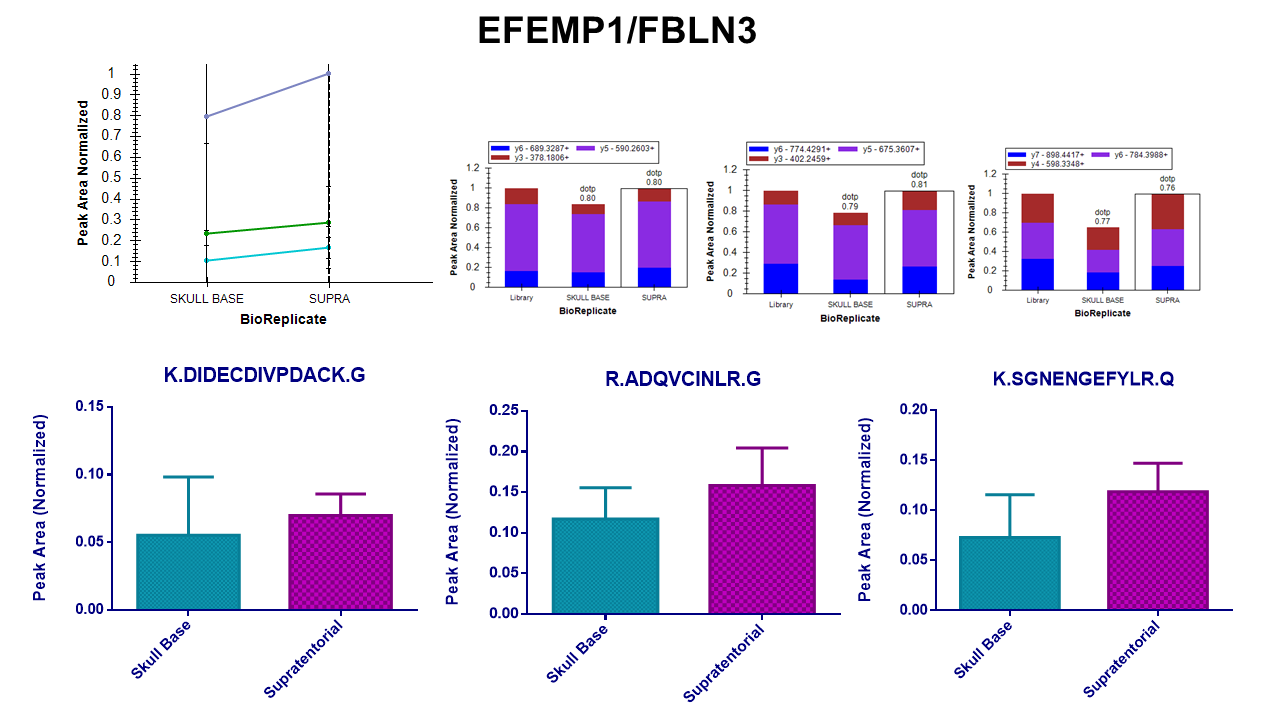

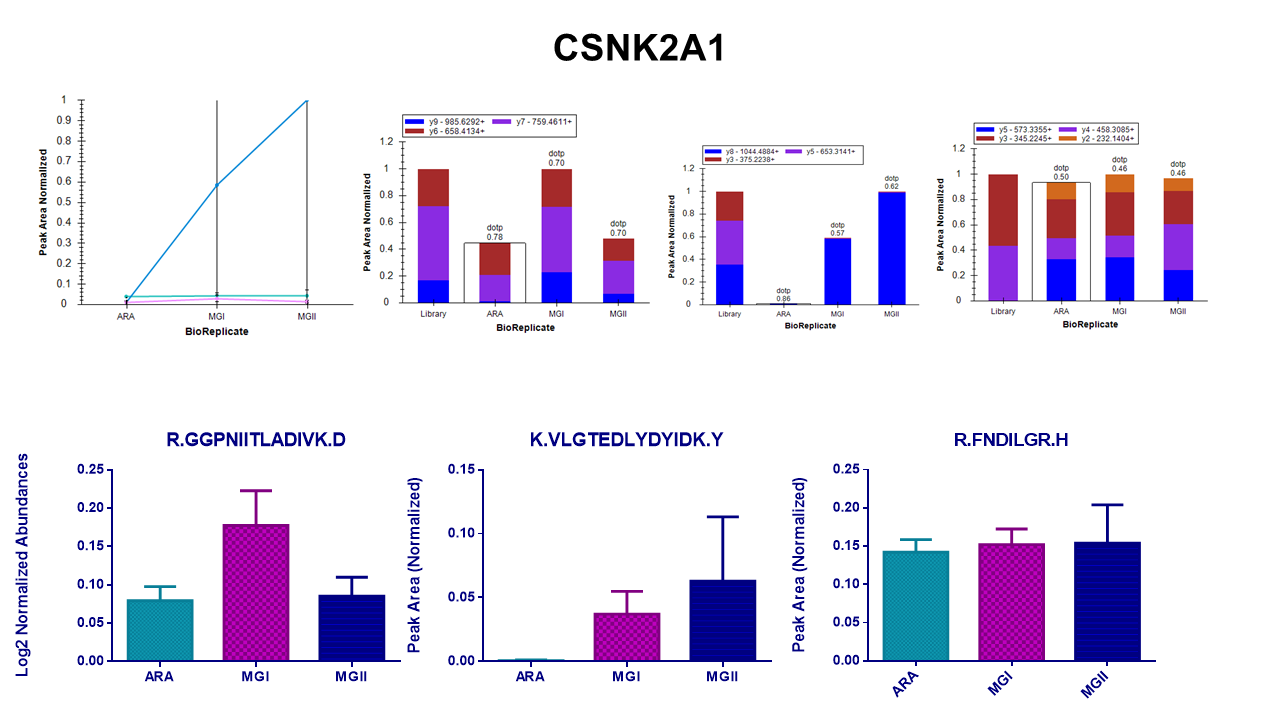

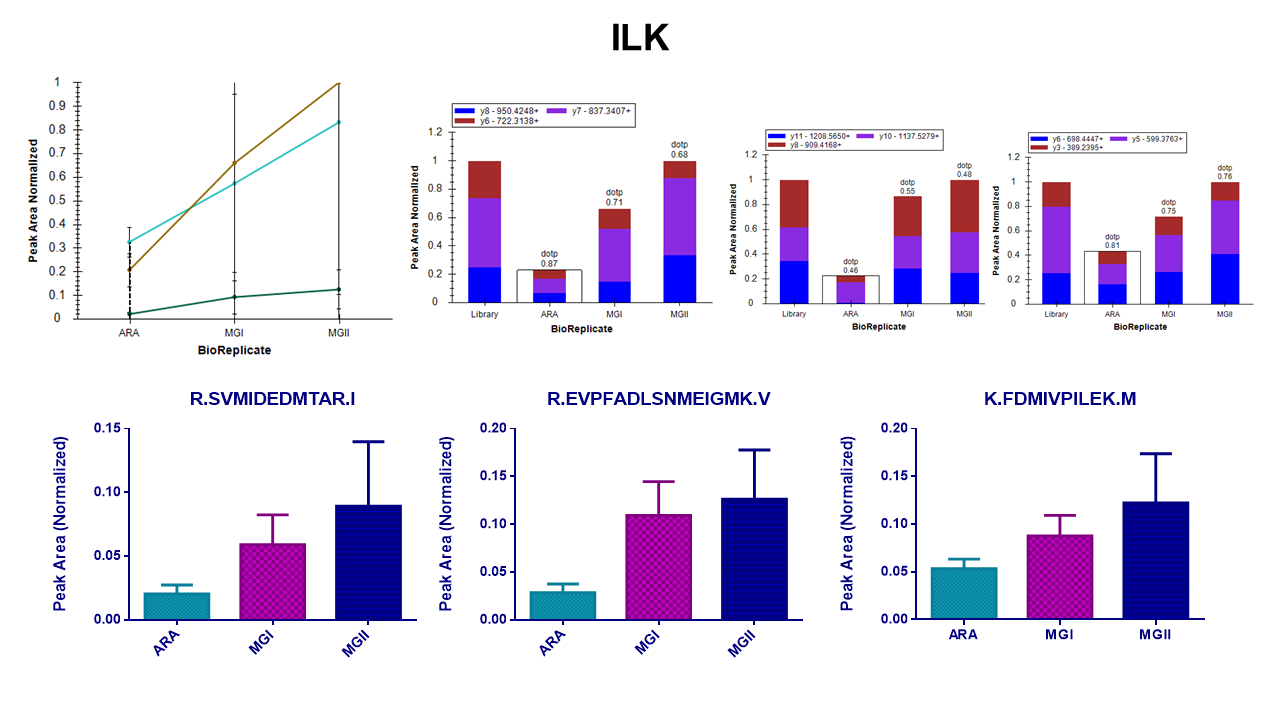

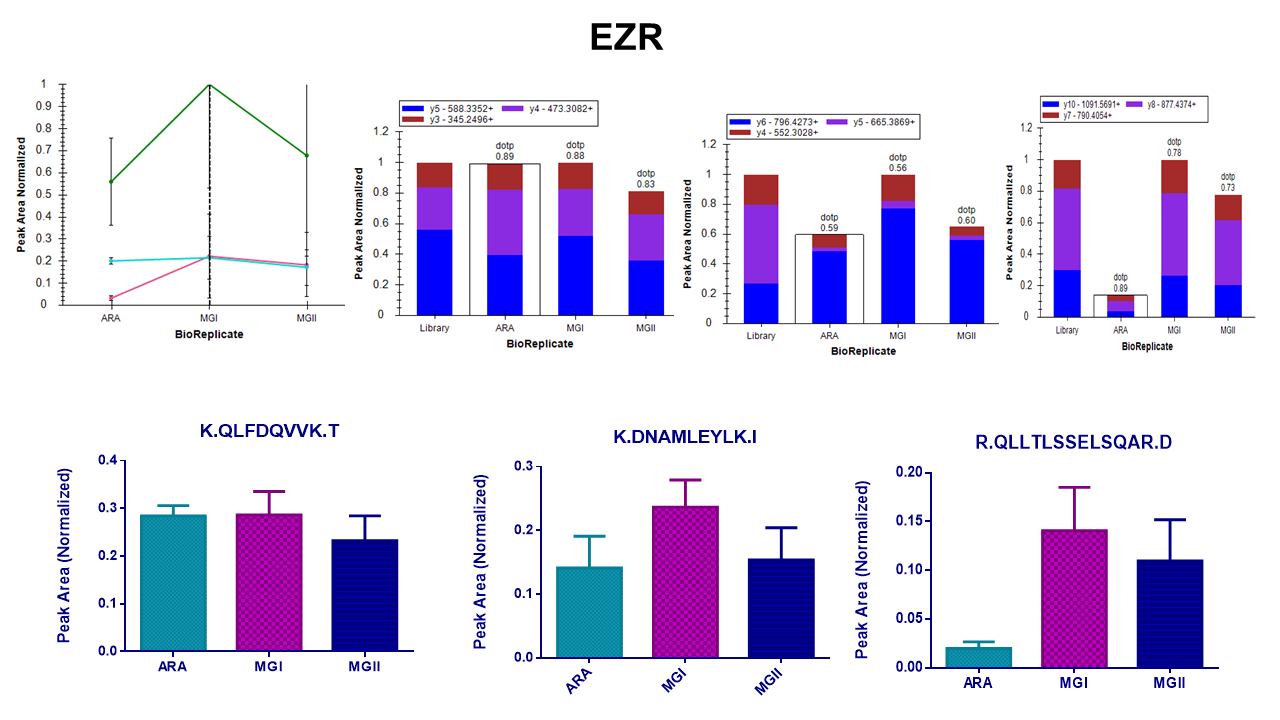

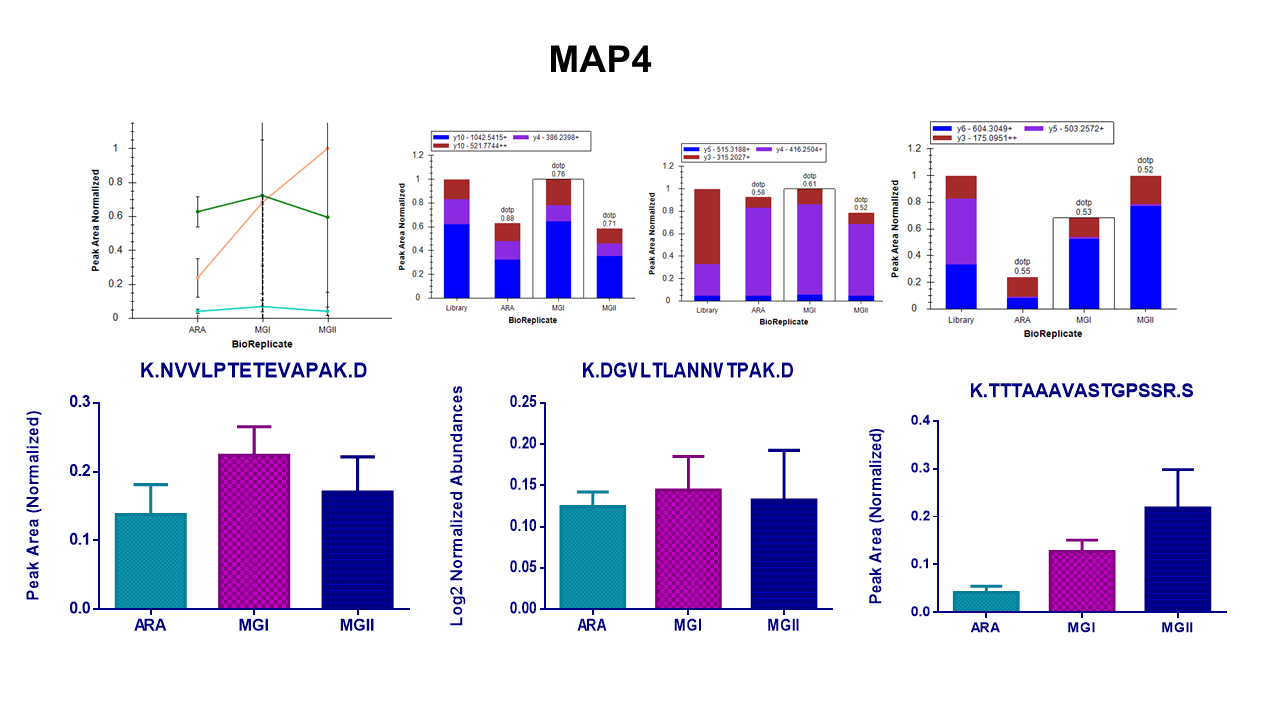

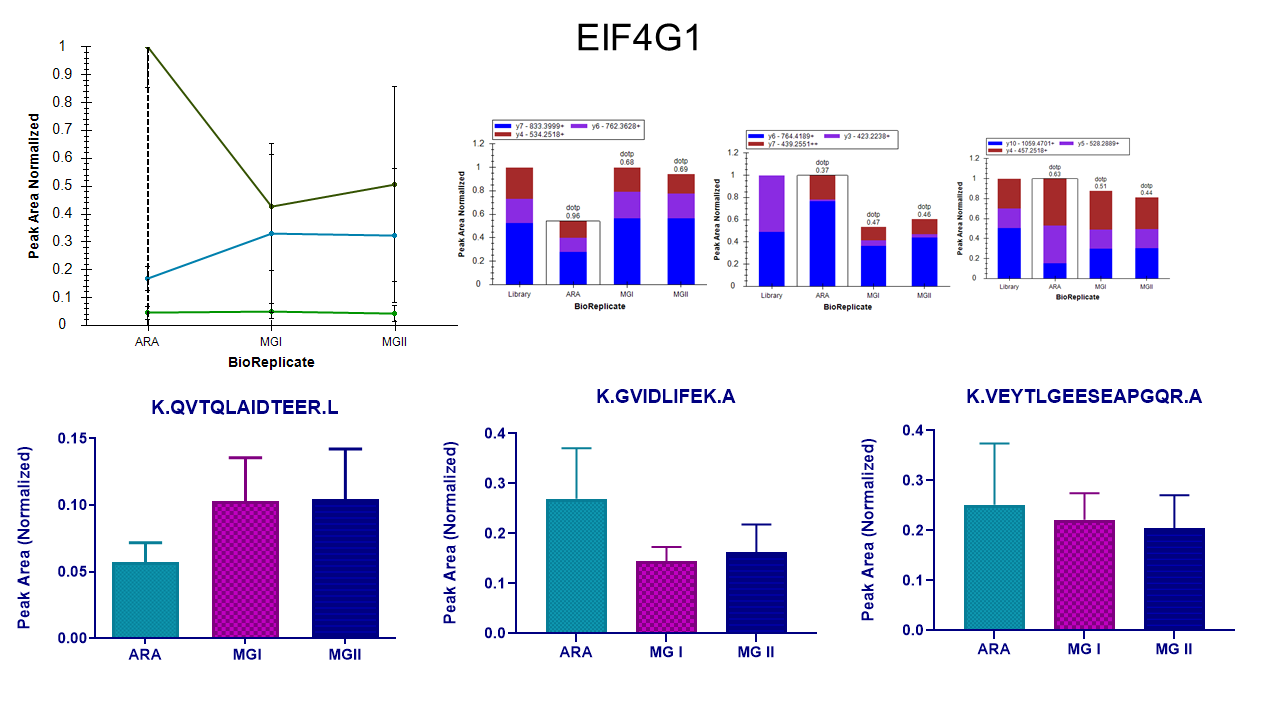

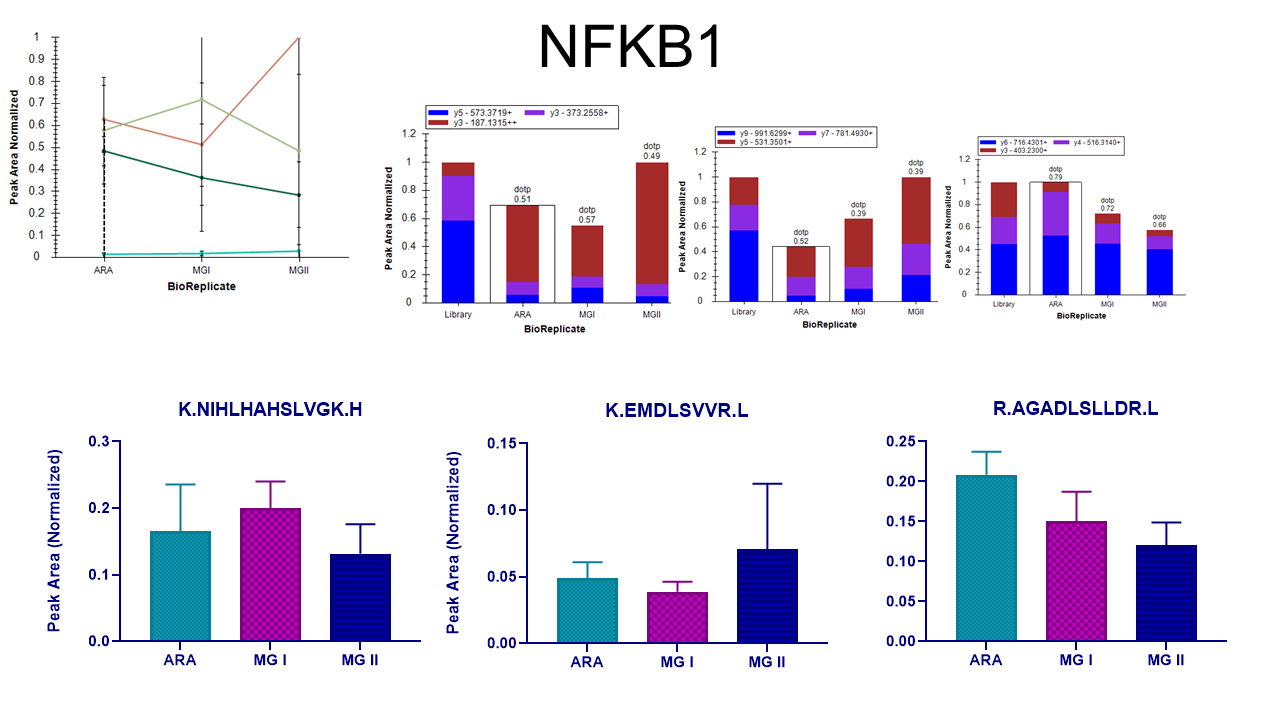


**Supplementary Figure 5: SRM Analysis outcomes in various grades of meningioma and non tumor controls**

**Supplementary Figure 6: Label free based mass spectrometry analysis enabled identification of several components of Integrin pathway, key candidates enumerated(p values ≤ 0.05) as tabulated below. Curated from Supplementary data table 2**

**Annexure to Supplementary Figure 6:**


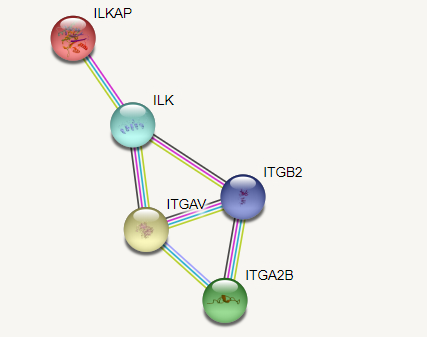
Table stating the datapoints for the key components of Integrin pathway and Interaction as procured from STRING DB®

| **Gene Symbol** | **MGI** | **MGII** | **p value** |
| --- | --- | --- | --- |
| **ITGAV** | **25.615** | **24.577** | **0.02456** |
| **ITGB2** | **25.881** | **25.363** | **0.00001** |
| **ITGA2B** | **23.999** | **23.668** | **0.01773** |
| **ITGA2** | **25.863** | **25.625** | **0.00044** |
| **ILKAP** | **23.264** | **19.161** | **0.02130** |
| **ITGAX** | **24.438** | **23.421** | **0.03862** |
| **ADAM17** | **22.397** | **23.553** | **0.01068** |
