# Supplementary material for: Comprehending Meningioma Signaling Cascades Using Multipronged Proteomics Approaches & Targeted Validation of Potential Markers
Source: Front Oncol. 2020 Aug 26;10:1600. doi: 10.3389/fonc.2020.01600 (PMC7482667; doi:10.3389/fonc.2020.01600)
Supplement: Supplementary file 11 [file Data_Sheet_2.docx]

Supplementary Material

**Multipronged proteomic analysis of meningiomas provide cues to the interplay of Integrin & PI3K-Akt pathway components**

Shuvolina Mukherjee^1^, Deeptarup Biswas^1^, Rucha Gadre^1^, Pooja Jain^2^, Nelofer Syed^3^, Julianna Stylianou^3^, Qingyu Zeng^3^, Anita Mahadevan^4^, Sridhar Epari ^5^, Prakash Shetty^6^, Aliasgar Moiyadi^6^, Graham Roy Ball^7^, Sanjeeva Srivastava^1*^

^1^Proteomics Lab, Department of Biosciences & Bioengineering, IIT Bombay, Mumbai, 400076, Maharashtra, India

^2^Centre for Integrative Systems Biology and Bioinformatics (CISBIO), Sir Ernst Chain Building, Department of Life Sciences, Imperial College London, London SW7 2AZ, UK

^3^Division of Brain Sciences, Department of Medicine, Imperial College London, Hammersmith Hospital Campus, Burlington Danes Building, London W12 0NN, UK

^4^Department of Neuropathology, Human Brain Tissue Repository (Brain Bank), NIMHANS, Bangalore- 5600029, India

^5^Department of Pathology, Tata Memorial Centre, Mumbai, Dr. E Borges Road, Parel, Mumbai - 400 012, India

^6^Department of Neurosurgery, Tata Memorial Centre, Mumbai, Dr. E Borges Road, Parel, Mumbai - 400 012, India

^7^School of Science and Technology, Nottingham Trent University, Clifton Lane, Nottingham, NG11 8NS, UK

*** Correspondence:**

Sanjeeva Srivastava

[sanjeeva@iitb.ac.in](mailto:sanjeeva@iitb.ac.in)

**Details of Supplementary Data Tables**

| **Data** | **Content** |
| --- | --- |
| **Supplementary Data 1** | **Clinical Information** |
| **Supplementary Data 2** | **Label-Free Proteomics Outcomes, Proteome Discoverer 2.0 Analysis** |
| **Supplementary Data 3** | **ANOVA Analysis & Grade-wise statistics** |
| **Supplementary Data 4** | **A. GSEA analysis of ANOVA pass proteins**  **B. Low grade vs High-Grade Interactions (ANN Analysis)** |
| **Supplementary Data 5** | **MGI vs MGII GSEA analysis** |
| **Supplementary Data 6** | **Phosphoproteomics data & Kinome Analysis Input files** |
| **Supplementary Data 7** | **Comparison with published proteomics datasets** |
| **Supplementary Data 8** | **RT_2_ PCR Analysis** |
| **Supplementary Data 9** | **A. Grade-specific proteins**  **B. Pathway component analysis** |

All Supplemtary files have been uploaded in google drive link due to large file size.

https://drive.google.com/open?id=1UXQ3lemv-XynDdpRMLyaiV02yaVxs0YF
